# Myo-inositol phosphate synthase expression in the European eel (*Anguilla anguilla*) and Nile tilapia (*Oreochromis niloticus*): effect of seawater acclimation

**DOI:** 10.1152/ajpregu.00056.2016

**Published:** 2016-06-01

**Authors:** Svetlana Kalujnaia, Neil Hazon, Gordon Cramb

**Affiliations:** ^1^School of Medicine, University of St. Andrews, St. Andrews, United Kingdom; and; ^2^School of Biology, University of St. Andrews, St. Andrews, United Kingdom

**Keywords:** *Anguilla anguilla*, *Oreochromis niloticus*, teleost, myoinositol phosphate synthase, myo-d-inositol 3-phosphate synthase, osmoregulation

## Abstract

A single *MIPS* gene (*Isyna1*/*Ino1*) exists in eel and tilapia genomes with a single myo-d-inositol 3-phosphate synthase (MIPS) transcript identified in all eel tissues, although two MIPS spliced variants [termed MIPS_(s)_ and MIPS_(l)_] are found in all tilapia tissues. The larger tilapia transcript [MIPS_(l)_] results from the inclusion of the 87-nucleotide intron between exons 5 and 6 in the genomic sequence. In most tilapia tissues, the MIPS_(s)_ transcript exhibits much higher abundance (generally >10-fold) with the exception of white skeletal muscle and oocytes, in which the MIPS_(l)_ transcript predominates. SW acclimation resulted in large (6- to 32-fold) increases in mRNA expression for both MIPS_(s)_ and MIPS_(l)_ in all tilapia tissues tested, whereas in the eel, changes in expression were limited to a more modest 2.5-fold increase and only in the kidney. Western blots identified a number of species- and tissue-specific immunoreactive MIPS proteins ranging from 40 to 67 kDa molecular weight. SW acclimation failed to affect the abundance of any immunoreactive protein in any tissue tested from the eel. However, a major 67-kDa immunoreactive protein (presumed to be MIPS) found in tilapia tissues exhibited 11- and 54-fold increases in expression in gill and fin samples from SW-acclimated fish. Immunohistochemical investigations revealed specific immunoreactivity in the gill, fin, skin, and intestine taken from only SW-acclimated tilapia. Immunofluorescence indicated that MIPS was expressed within gill chondrocytes and epithelial cells of the primary filaments, basal epithelial cell layers of the skin and fin, the cytosol of columnar intestinal epithelial and mucous cells, as well as unknown entero-endocrine-like cells.

the cyclic polyol, inositol, is a cellular metabolite with universal existence in all organisms from bacteria to humans. Inositol and its chemically modified congeners (e.g., phosphorylated derivatives such as inositol mono-, bis-, and tris-phosphate) are involved in many intracellular processes, such as hormonal signaling, regulation of gene expression, cell growth, membrane biogenesis and trafficking, protein stabilization, and cellular osmoregulation (8, 9, 13, 14, 16, 18). Although inositol's role as a compatible intracellular osmolyte has been well documented in salinity adaptation in plants and in volume regulation in mammalian renal epithelia, it is only recently that its importance in osmoregulation in euryhaline teleosts, such as the eel (*Anguilla anguilla*) and tilapia (*Oreochromis niloticus* and *Oreochromis mossambicus*), has been investigated (15, 24, 25, 38, 39, 44). Intracellular inositol levels have been shown to increase in a variety of tissues to help compensate for the increases in extracellular osmolality associated with the movement of fish from freshwater (FW) to seawater (SW) or to hyper-SW environments (10, 24, 25). The osmolyte is particularly important in epithelial cells that are directly exposed to the external SW environment, such as in the skin, fin, and gill. Inositol can be synthesized de novo from glucose 6-phosphate (G 6-P) by the sequential actions of two enzymes, myo-d-inositol 3-phosphate synthase (MIPS), which converts G 6-P to inositol 3-phosphate (IP) and inositol monophosphatase (IMPA), which dephosphorylates IP to free inositol. IMPA can dephosphorylate both the inositol 3-phosphate that is generated from MIPS activity and also any inositol 1-phosphate that is produced from cell signaling and the turnover of membrane inositol phospholipids (36). Alternatively, in animal cells, the cyclic alcohol can be sequestered as the result of the actions of two types of membrane transporter, the sodium- and the proton-dependent myo-inositol transporters (abbreviated as SMIT and HMIT, respectively) (41), which use the transmembrane sodium or proton gradients to accumulate the metabolite to concentrations of up to 1,000 times that found in the extracellular environment. Adaptation of euryhaline fish to SW or hyper-saline environments has been reported to result in an increase in expression of IMPA in a number of tissues from the eel (24, 25), killifish (45), and tilapia (15, 24, 25, 39, 44). Although only two *IMPA* genes have been reported in most vertebrate species, teleost-specific tandem and whole genome duplication events have enabled fish the potential to harbor up to six different IMPA transcripts (24). Four *IMPA* genes have been identified in both the eel (*A. anguilla*) and tilapia (*O. niloticus*). Although these IMPA isoforms exhibit species-specific tissue expression profiles, the *Impa1.1* gene appears to be the only isoform that displays significant salinity-sensitive regulation of expression in the eel (24, 25) and two species of tilapia, *O. niloticus* (24) and *O. mossambicus* (39). It has been suggested that the amino acid sequence of this isoform has been selected throughout evolution as a more salt-tolerant protein with optimal enzyme activities found at the higher ionic strengths and pHs found in the tissues of marine teleosts (44). In most organisms, MIPS is considered to be the rate-limiting enzyme of the inositol biosynthetic pathway (21) and, unlike IMPA, only one gene (termed *Isyna1* or *Ino1*) is found in the genome databases of most vertebrates, although tissue-specific splice variants have been reported in a number of species. The extent to which *MIPS* may be regulated at transcriptional and translational levels following SW acclimation in euryhaline fish has still to be fully determined. Two MIPS splice variants are expressed in tissues of the Mossambique tilapia (*O. mossambicus*) with exposure of fish to hyper-saline conditions (90 ppt), resulting in an upregulation in the expression of mRNAs for both forms in the brain (15) and the gill (39). It is unknown whether similar salinity-sensitive splice variants are present in all euryhaline teleosts. Current evidence suggests that increases in the expression and/or activities of both IMPA and MIPS enzymes are essential for the accumulation of inositol and the survival of Mossambique tilapia, as they acclimate to marine/hyper-saline environments, however, it is unknown whether this extends to other euryhaline teleosts, including evolutionary divergent species, such as the eel. In this article, we report the effects of SW transfer on MIPS expression and tissue distribution in the European eel (*A. anguilla*) and the Nile tilapia (*O. niloticus*).

## MATERIALS AND METHODS

### 

#### Fish.

Adult migratory female silver eels (*A. anguilla*; 300–450 g) and mixed-sex adult red hybrid tilapia (*O. niloticus*, 300–400 g) were obtained from local suppliers and maintained on a 12:12-h light-dark cycle either at ambient temperature (7–10°C eels) or at 24°C (tilapia), as previously described (24, 25). Eels were acutely transferred from FW to FW or FW to SW by gradually emptying and refilling 300-liter tanks over a 4-h period and maintained for a further 3 wk before use in experiments. Tilapia were gradually acclimated to full SW over a 4-wk period, as described previously (24), and fish were sampled 10 and 8 wk after transfer to 50% or 100% SW, respectively. Eels were not fed during the experimental period (eels will not feed when water temperatures are below 10°C), but tilapia were fed with a standard chow (20 g/kg wet weight/day) until 48 h before killing and tissue removal, as detailed previously (24, 25). All protocols were conducted in accordance with the Animals Scientific Procedures Act, 1986, under Home Office Project License No. 603805, and all experiments were approved by the University of St. Andrews Animal Welfare and Ethics Committee. All tissues were either immediately frozen and stored at −80°C for RNA and protein extraction or fixed for 24 h in 4% paraformaldehyde in PBS before being washed in PBS and step-wise dehydrated in ethanol before embedding in paraffin. Tissues were processed for immunohistochemistry, as detailed previously (29, 34).

#### Cloning and sequencing.

Total RNA from selected tissues was treated with DNase and reverse transcribed for use in PCR experiments (24, 25). Standard PCR reactions included 1 μl template cDNA (diluted 1:2–1:10), 1 μl each of sense and antisense primers (5 μM) in a total volume of 20 μl comprising 1 × reaction buffer containing 2 mM MgCl_2_ (Biogene, Huntingdon, UK), 250 nM dNTPs (Promega, Southampton, UK) and 1.25 units Taq DNA polymerase Gold (Biogene). PCR cycles routinely included a 2-min denaturation step at 92°C followed by 30 cycles of 94°C (10 s) 60°C (20 s), and 72°C (1 min/kbp), and a final 10-min extension at 72°C. Oligonucleotide primers (Eurofins MWG Operon, Ebersberg, Germany) for eel and tilapia MIPS and three “reference” genes, large acidic ribosomal phosphoprotein (*Rpl-P0*), cyclophilin A (*CycA*), and phosphoglycerate kinase 1 (*Pgk1*), used for expression normalization (Table 1), were designed on the basis of sequence information deposited in nucleotide databases [NCBI, Ensembl, and the eel genome library: http://www.zfgenomics.org/sub/eel (35)]. The sequences of all amplified cDNA fragments were confirmed by first cloning into the pCR4 vector using the TOPO-TA cloning system (Life Technologies, Paisley, UK) and then sequenced using standard cycle sequencing (Big Dye, Applied Biosystems, Cheshire, UK) with electrophoretograms and sequences analyzed using 4Peaks (Softonic International) and GeneJockey II (Biosoft, Cambridge, UK) software (24). The full-length eel MIPS sequence was deposited in the EMBL GenBank under the accession no. LT159970 together with eel CycA (accession no. LT159971) and a partial cDNA of eel Pgk1 (accession no. LT159972).

**Table 1.** Primer sequences used for quantitative RT-PCR

Species	Gene	Sense Primer	Antisense Primer	Amplicon Size, bp
*Anguilla anguilla*	MIPS	CTGTGTGGTGATCAAGTATGTGCCGTAC	GCTGAGCAGCGACAGCACGCTGTG	187
	Rpl-P0	TGAAGTCTTGAGCGATGTGCA	GGAGAAGGGCGAGATGTTCAG	97
	CycA	CGTCCAAAACAGAATGGCTGGACGGC	CGCACTCCATGACGGTGATCCTCTTG	138
	Pgk1	GCGACCACCGGCACCGCCAC	CTGCTTGGCTCTGCCTACCGCTTC	114
*Oreochromis niloticus*	MIPS(s)	GAGCAGCTGCGACCATACATGAGC	CTCTAATTCGCTCCATCTGCTCCGC	139
	MIPS(l)	GAGCAGCTGCGACCATACATGAGC	CAGCTCTAATTCGCTCCATCTGGAAGATC	165
	Rpl-P0	GCCATCCGTGGCCATCTGGAG	CGGGCAGCTGCAGGTACCTTG	146
	CycA	CAGCCTCAACTGACTGGCTGAATGGG	CACCACAGTCAGCAATGACAATCTTGGC	141
	Pgk1	GCCGCCACCGGCACTGCAACAG	CTTGGCCCTGCCGACCGCCTC	111

#### Bioinformatics and comparative modeling.

Amino acid identity and similarity analyses for known MIPS protein sequences were calculated using MatGat2.01 software (4). For phylogenetic analysis, amino acid sequences were obtained from available genome databases (Table 2) and aligned using Clustal Ω software. Phylogenetic trees were constructed (Mega 5 software) using the neighbor-joining method (40). The statistical reliability was assessed by bootstrap analyses with 1,000 replications.

**Table 2.** Accession numbers for MIPS isoforms/spliceforms used in phylogenetic analysis and reference genes used for data normalization

Species	Common Name	EMBL/NCBI Accession Number
*Accession Numbers for MIPS ESTs*		
*Aedes aegypti*	Yellow fever mosquito	XM_001655948.1
*Anguilla anguilla*	European eel	LT159970
*Anolis carolinensis*	Lizard	XM_003230040.2
*Apis florea*	Honey bee	XM_003692729.1
*Bos taurus*	Cow	NM_001046032.2
*Caenorhabditis elegans*	Nematode	NM_064098.5
*Callorhinchus milii*	Australian ghostshark	JX053296.1
*Canis lupus*	Dog	XM_533872.4
*Chrysemys picta bellii*	Painted turtle	XM_005278998.2
*Culex quinquefasciatus*	House mosquito	XM_001848293.1
*Cyprinus carpio*	Common carp	JF836164.1
*Drosophila melanogaster*	Common fruit fly	AF071104.1
*Ficedula albicollis*	Collared flycatcher	XM_005060186.1
*Gadhus morhua*	Atlantic cod	ENSGMOG00000012071
*Gallus gallus*	Chicken	XM_015300145.1
*Gasterosteus aculeatus*	Three-spined stickleback	ENSGACG00000009858
*Homo sapiens variant 1*	Human	NM_016368.4
*Homo sapiens variant 2*	Human	NM_001170938.1
*Homo sapiens variant 4*	Human	NM_001253389.1
*Latimeria chalumnae*	Coelacanth	ENSLACG00000017582 + ENSLACG00000000593
*Mus musculus*	House mouse	NM_023627.1
*Oreochromis mossambicus*	Mozambique tilapia	DQ465381.1
*Oreochromis niloticus v1*	Nile tilapia	XM_003442813.2
*Oreochromis niloticus v2*	Nile tilapia	XM_005477233.1
*Oryzias latipes*	Japanese rice fish	ENSORLG00000014091
*Petromyzon marinus*	Sea lamprey	ENSPMAG00000005085
*Pseudotropheus zebra*	Maylandia zebra	XM_004540270.1
*Pundamilia nyererei*	Mwanza Gulf cichlid	XM_005723690.1
*Saimiri boliviensis*	Squirrel monkey	XM_003942285.1
*Salmo salar*	Salmon	NM_001140330.1
*Sarcophilus harrisii*	Tasmanian devil	XM_003760772.1
*Strongylocentrotus purpuratus*	Purple sea urchin	XM_003728376.1
*Takifugu rubripes*	Japanese puffer fish	ENSTRUG00000015897
*Tetraodon nigroviridis*	Green spotted puffer fish	ENSTNIG00000017194
*Xenopus laevis*	African clawed frog	NM_001086071.1
*Xenopus tropicalis*	Western clawed frog	NM_001126513.1
*Xiphophorus maculatus*	Southern platyfish	ENSXMAG00000010138
*Accession Numbers for Cyclophilin A, Pgk1, and Rpl-P0 cDNAs*		

	Gene	NCBI Accession Number
		
*Anguilla anguilla*	Cyclophilin A	LT159971
	Pgk1	LT159972
	Rpl-P0	AY763793.1
*Oreochromis niloticus*	Cyclophilin A	XM_003439486.3
	Pgk1	XM_003451451.3
	Rpl-P0	XM_003441555.2

#### Real-time quantitative PCR (RT-qPCR).

Total RNA samples (2 μg) from each tissue were DNase-treated, reverse-transcribed (M-MLV-RT, RNase H-, from Promega, Southampton, UK), and 97-187-bp fragments from the coding regions of MIPS, Rpl-P0, CycA, and Pgk1 transcripts were amplified using PerfeCTa SYBR Green FastMix (Quanta Biosciences, VWR, UK) (see Table 1 for primer sequences). PCR reactions comprised 8 μl of 1:16 dilution of cDNA, 1 μl each of sense and antisense primers (5 μM), and 10 μl of 2× SYBR Green Mix. Using pooled aliquots of cDNA from all samples, efficiency curves for all primer sequences used in experiments indicated amplification efficiencies of 85–105% over a 4-log scale. The expression of mRNAs for large acidic ribosomal phosphoprotein (*Rpl-P0*), cyclophilin A (*CycA*), and phosphoglycerate kinase 1 (*Pgk1*) were all found to be invariable across most tissues tested from FW- and SW-acclimated eels and tilapia (coefficients of variation calculated for Ct values from combined FW and SW samples within each tissue for both species ranged from 2.4 to 8.6% for *Rpl-P0*), and therefore, *Rpl-P0* was routinely used as the reference gene for data normalization in both species. Coefficients of variation for Ct values for MIPS primers used within species and experimental groups ranged from 3.4% (FW tilapia gill) to 13.6% (SW tilapia kidney). The relative abundance of MIPS mRNAs was determined using the standard delta Ct method [i.e., 2̂^−(Ct MIPS−Ct Rpl−P0)^] to obtain normalized relative expression values. Expression values for each cDNA sample were determined in duplicate on the same RT-qPCR reaction plate along with template-negative and reverse transcriptase-negative controls. Results were analyzed using the Applied Biosystems 7300 System SDS detection software (Life Technologies, Paisley, UK).

#### Protein preparation and Western blot analysis.

Supernatant fractions from tissue homogenates were prepared and processed for Western blot analysis as described previously (24). Protein concentrations in all fractions were determined by the Bradford method (3) (Bio-Rad Laboratories, Hercules, CA), following the manufacturer's protocol. Denatured proteins (10 μg) were separated by NuPAGE (Life Technologies) gradient (4–12%) PAGE blotted onto polyvinylidene difluoride membranes (VWR, Leicestershire, UK) and sequentially probed with a mouse polyclonal antiserum raised against an internal region of the human MIPS protein (anti-ISYNA1; 1:2,000 dilution, Abnova, Heidelberg, Germany) and then stripped and reprobed with an affinity-purified mouse monoclonal antibody raised to human alpha-tubulin (final concentration 0.4 μg/ml) and to assess protein loading, as described previously (24). The anti-hISYNA1 antibody was raised to amino acids 331–430 of the human sequence, and this region exhibited 87% and 86% identity and 93% and 94% similarity with the eel and tilapia sequences, respectively. Blots were also conducted using two additional heterologous antibodies to validate antibody cross reactivity with tilapia and eel MIPS. Selected blots were probed with a rabbit anti-hISYNA1 polyclonal antibody raised to amino acids 141–314 (Abcam ab118241; final dilution 1/1,000) and a mouse anti-hISYNA1 monoclonal antibody raised to amino acids 219–253 (Santa Cruz Biotechnology; C-8, sc-377245; final dilution 1/200). Epitopes are highlighted in Supplemental Fig. S1. The amino acid identities/similarities of the human and fish sequences in these regions were 73.6/89.7% and 73.6/87.4% for the Abcam antibody and 83.6/98.2% and 85.4/98.2% for the Santa Cruz antibody for tilapia and eel, respectively. The binding of primary antibodies was detected using HRP-conjugated anti-mouse or anti-rabbit IgG secondary antibodies (Sigma Aldrich; 1:20K dilution), and the conjugated secondary antibodies were visualized using the ECL detection method (SuperSignal West Femto ECL, ThermoFisher Scientific, Loughborough, UK) (31, 34).

#### Immunohistochemistry.

Immunohistochemical localization of MIPS was conducted on fixed tissue samples (gill, skin, fin, and intestine) from FW- and SW-acclimated eels and tilapia using anti-MIPS (Abnova anti-hISYNA1) primary antisera (1:400 final dilution) and visualized using a 1:800 final dilution of Dy-Light 488-conjugated goat anti-mouse IgG secondary antibody (Abcam, Cambridge, UK), as described previously (24, 29, 34). Sections were counterstained with DAPI to allow detection of cell nuclei. Sections were viewed with a fluorescence microscope (Zeiss Axioplan, Welwyn Garden City, Hertfordshire, UK) equipped with the appropriate filters, and images were collected and analyzed using Zeiss Axiovision Software. Control sections incubated in the absence of primary antisera were run routinely.

#### Statistical analyses.

Values are presented as means ± SD or means ± SE. Statistical significance within the RT-qPCR and Western blot analyses was determined using a nonpaired Students *t*-test or by two-way ANOVA followed by Fisher's paired least significant difference post hoc analysis of significance (StatView 4.01 software; Abacus Concepts, Berkeley, CA). Significant differences are labeled as *P < 0.05, **P < 0.01, and ***P < 0.001.

## RESULTS

### 

#### Sequence, phylogenetic analysis, and tissue distribution of MIPS.

**Fig. 1.**
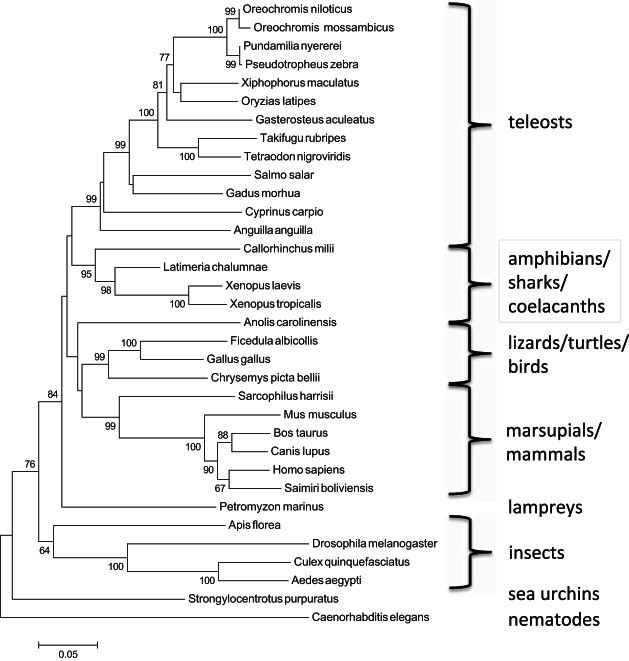
Phylogenetic analysis of myo-inositol synthase (MIPS). The phylogenetic tree was constructed using Mega5 software using the neighbor-joining method. The statistical reliability of individual nodes of the tree was assessed by bootstrap analysis with 1,000 replicates. NCBI/EMBL accession numbers are given in Table 2.

A single *MIPS* gene is annotated in both the eel and tilapia genome databases. In the Nile tilapia, two 5′UTR transcript variants are predicted in the database (see Table 2), although coding and 3′UTRs are identical, resulting in a putative 553 amino acid protein with molecular weight of 60.6 kDa. In the European eel genome database, BLAST analyses revealed the presence of a single *MIPS* analog on scaffold 7641, generating a mature protein of 60.4 kDa. The alignment of MIPS amino acid sequences from eel, tilapia, and all currently available teleost species along with selected sequences from other phyla are presented in Supplemental Fig. S1, and the phylogenetic analysis is shown in Fig. 1. As expected, the two species of tilapia (*O. niloticus* and *O. mossambicus*) and two other African cichlids (*P. nyererei* and *P. zebra*) exhibit the highest levels of amino acid homology (>96%; Supplemental Table S1); however, the alignment and phylogeny emphasize the high levels of amino acid similarity that exist between all species. Within the teleosts, amino acid sequence homologies were generally greater than 75%, with homology across all vertebrate species greater than 60%. The high-sequence homology also extends to invertebrates where sequence identities to most vertebrates, including mammals, are generally greater than 50%. Perhaps somewhat surprisingly, the amphibians appear to exhibit closer alignment to the coelocanths than the lizards and turtles. The MIPS sequence reported for *O. mossambicus* differs from that of *O. niloticus*, and indeed from all other species with a 114 nucleotide deletion from exon 7 and a 9 nucleotide repeat inserted in exon 3, resulting in a slightly smaller protein of 518 amino acids and a theoretical molecular weight of 57.2 kDa. Accession numbers for all sequences are presented in Table 2.

Species-specific primers were used to amplify and confirm the coding sequences of the expressed MIPS constructs in both eel and tilapia (results not shown). In tilapia, PCR analyses identified the presence of two transcripts in all tissues tested. Sequence analysis determined that the larger transcript incorporated the 87-bp intron between exons 5 and 6 of the tilapia gene, which would generate a 29 amino acid insert resulting in a translated mature protein with molecular weight of 64.5 kDa. This insert also contained potential additional phosphorylation sites to the many already predicted for this protein (Supplemental Fig. S1). Screening of the eel genome identified a similar intronic region that was the equivalent to the tilapia insert. Although the intron contained no stop codons nor frame shifts, there was no sequence homology to the tilapia intron, and transcripts containing this insert could not be detected by PCR in any tissue tested from FW- or SW-acclimated fish (results not shown).

**Fig. 2.**
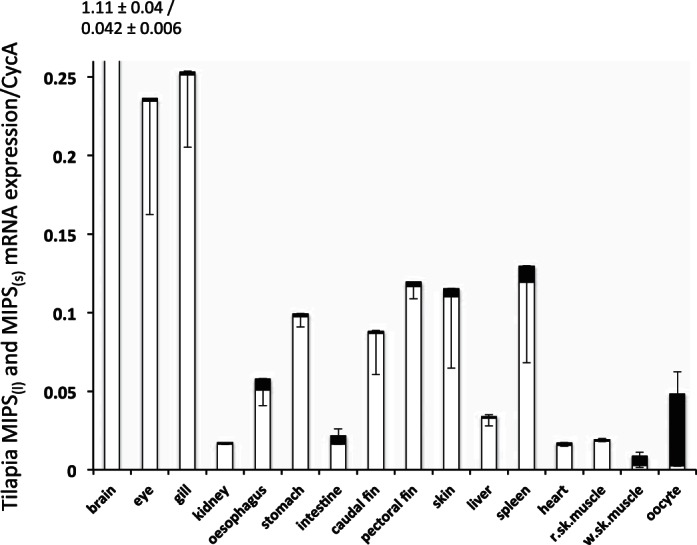
Relative tissue expression of tilapia myo-inositol phosphate synthase_(l)_ MIPS_(l)_ (black bar) and MIPS_(s)_ (open bar) mRNAs. Complementary cDNA was synthesized from tissue total RNA pooled from at least three freshwater (FW) tilapia. Values represent the means ± SD of three observations of MIPS_(l)_ and MIPS_(s)_ expression. Expression values are relative to the expression of cyclophilin A (CycA) mRNA for that tissue. In the brain, the expression value for MIPS_(s)_ exceeded the ordinate scale, and this value along with that of MIPS_(l)_ is printed above the bar. Negative error bars indicate the error in MIPS_(s)_, and positive error bars indicate the error in MIPS_(l)_.

**Fig. 3.**
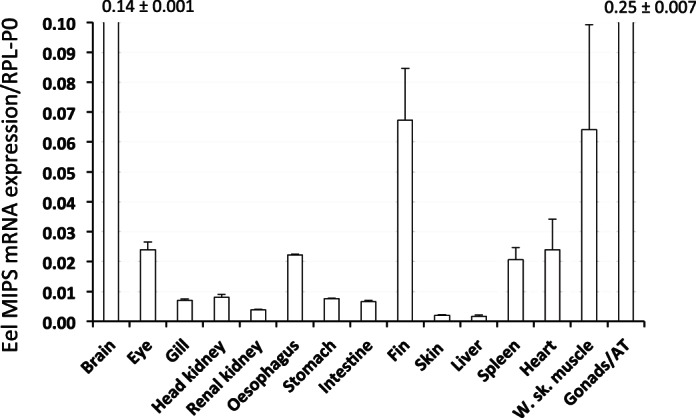
Relative tissue expression of eel MIPS mRNA. Complementary cDNA was synthesized from tissue total RNA pooled from at least three FW eels. Values represent the means ± SD of three observations of MIPS expression. Expression values are relative to the expression of large acidic riboprotein-P0 (Rpl-P0) mRNA for that tissue. In brain and the combined gonad/adipose tissues, the expression value for MIPS exceeded the ordinate scale, and these values are printed above the bar.

RT-qPCR indicated that in FW-acclimated tilapia the expression of the larger splice-variant, MIPS_(l)_, although present in all tissues investigated, was generally very low, and much less than the smaller splice variant, MIPS_(s)_, with the exception of white skeletal muscle and oocytes where the larger splice-variant predominated (Fig. 2). The relative expression of both transcripts across all tissues was normalized using CycA, as mRNA for Rpl-P0 could not be detected in oocytes (C_t_ > 32). In tilapia, the highest expression of MIPS_(s)_ was in the brain followed by similar levels in gill and eye with lower levels in the other tissues. In the FW-acclimated eel, the single MIPS transcript was abundant in all tissues with the highest levels in the combined gonad/adipose tissue samples followed by the brain, fin, and white skeletal muscle (Fig. 3). Lower, but substantial, levels were found in all other tissues.

#### Effect of salinity transfer on expression of MIPS in the eel and tilapia.

**Fig. 4.**
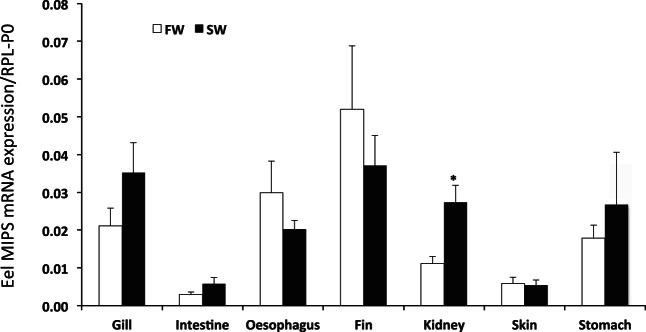
Relative expression of MIPS mRNA in tissues isolated from freshwater (FW)- and seawater (SW)-acclimated eels. The relative expression was calculated from Ct values obtained for MIPS amplifications from selected tissues taken from FW- (open bars) and SW- (black bars) acclimated fish, normalized to Rpl-P0 mRNA expression. Results are expressed as means ± SD of duplicate measures taken from tissue samples extracted from six fish. *P < 0.05.

**Fig. 5.**
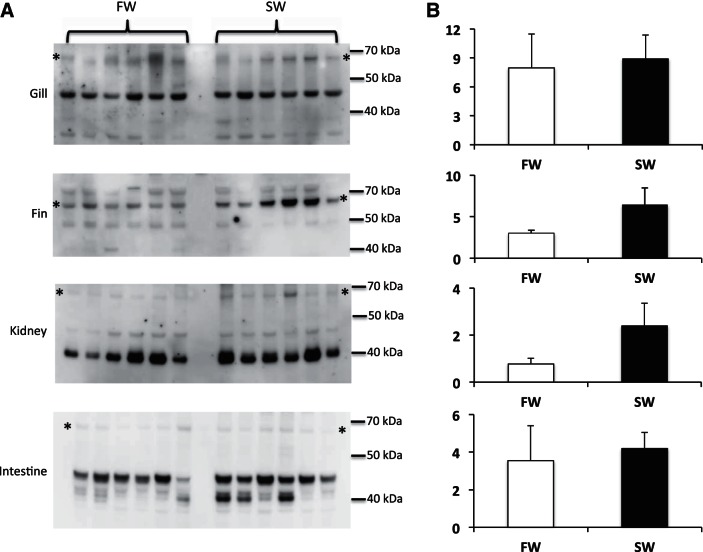
Western blot analyses of MIPS protein expression in eel tissues. The blots show the relative abundance of MIPS-immunoreactive proteins in gill, fin, kidney, and intestine. Tissue supernatant samples (10 μg protein) were blotted from each tissue and probed with an anti-hMIPS antibody (Abnova), as described in materials and methods. Blots (*A*) were imaged using a Fuji LAS3000 Image Reader, and all protein bands were quantified using AIDA data analysis software (Raytest, Straubenhardt, Germany), with the quantified results for the 67-kDa protein (designated by * in *A*) presented as means ± SE (*B*); *n* = 6. *P < 0.05.

Transfer of eels to SW resulted in no significant change in MIPS mRNA expression in all tissues tested, with the exception of the kidney, where a small but significant 2.5-fold increase was found (Fig. 4). Western blots of supernatant samples taken from FW- and SW-acclimated eels resulted in a range of tissue-specific immunoreactive proteins being detected with the Abnova anti-hISYNA1 antiserum (Fig. 5*A*). Although all tissues exhibited an immunoreactive protein of ∼67 kDa, stronger immunoreactive protein bands were found at 45 kDa in gill and intestine and 40 kDa in the kidney. Although the Santa Cruz C8 anti-hISYNA1 antibody failed to detect any immunoreactive protein in any eel tissue, the Abcam anti-hISYNA1 antibody identified similar lower-molecular-weight proteins in the gill, kidney, and intestine (results not shown). No matter which antibody was used, the Western blots failed to detect any immunoreactive protein band with the calculated theoretical molecular weight of eel MIPS (60.4 kDa). MIPS proteins from many species are known to run at higher molecular weights on denaturing gels compared with that calculated from their amino acid sequences (32, 33, 42), suggesting that the 67-kDa protein may be MIPS. In addition, all antibodies used detected a 68-kDa immunoreactive protein presumed to be human MIPS in a human retinoblastoma cell extract (Y-79, Abnova) (Fig. 8), even though the theoretical molecular weight based on the human MIPS sequence is 61 kDa. Tissue-specific COOH-terminal truncated forms with much lower molecular weight have also been reported in the rat (37, 42). Although the identities of the eel immunoreactive proteins remain to be determined, quantification of all immunoreactive proteins detected in each tissue revealed there was no significant difference in abundance between FW- and SW-acclimated fish. The quantified values for the 67-kDa species in each tissue, as detected by the Abnova antibody, are indicated in Fig. 5*B*.

**Fig. 6.**
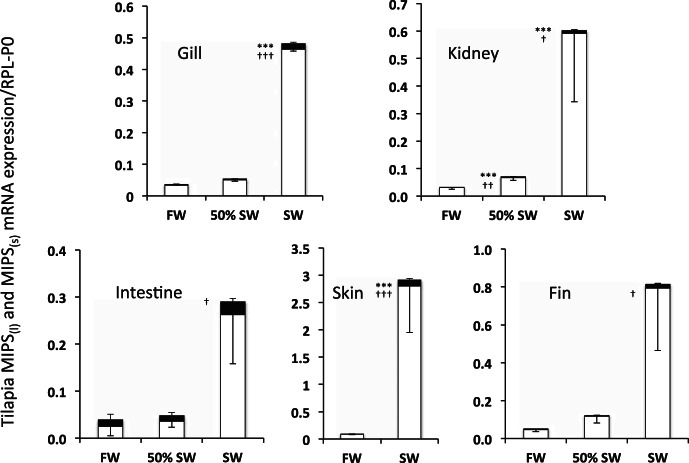
Relative expression of MIPS_(l)_ and MIPS_(s)_ mRNAs in tissues isolated from tilapia acclimated to freshwater (FW), 50% SW, and full seawater (SW). The relative expression was calculated from Ct values obtained for MIPS_(l)_ (black bars) and MIPS_(s)_ (open bars) amplifications from selected tissues taken from FW-, 50% SW-, and SW-acclimated fish, normalized to Rpl-P0 mRNA expression. Results are expressed as means ± SD of duplicate measures taken from tissue samples extracted from six fish. Negative error bars indicate the error in MIPS_(s)_, and positive error bars indicate the error in MIPS_(l)_. ***P < 0.001 compared with FW MIPS_(l)_ and †*P* < 0.05, †††*P* < 0.001 compared with FW MIPS_(s)_.

**Fig. 7.**
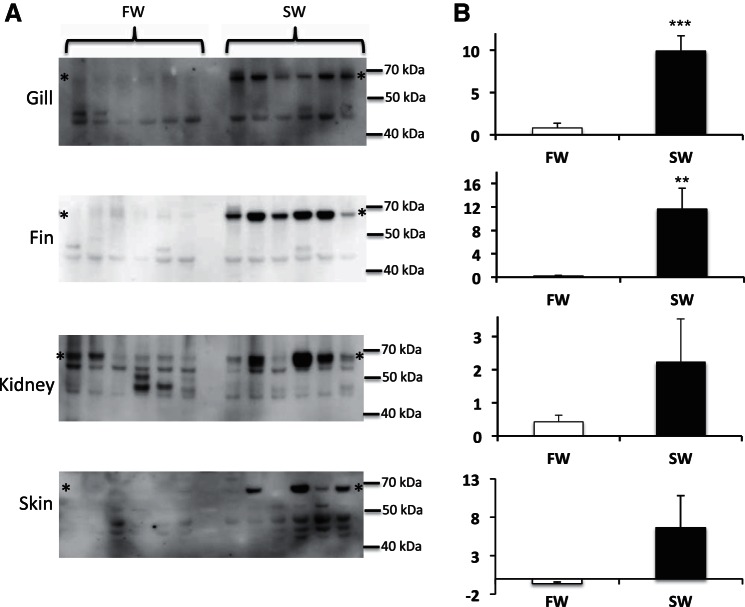
Western blot analyses of MIPS protein expression in tilapia tissues. The blots show the relative abundance of MIPS-immunoreactive proteins in gill, fin, kidney, and intestine. Tissue supernatant samples (10 μg protein) were blotted from each tissue and probed with an anti-hMIPS antibody (Abnova), as described in materials and methods. Blots (*A*) were imaged using a Fuji LAS3000 Image Reader, and all protein bands quantified using AIDA data analysis software (Raytest, Straubenhardt, Germany) with the quantified results for the major 67-kDa protein (designated by * in *A*) presented as means ± SE (*B*); *n* = 6. **P < 0.01, ***P < 0.001.

**Fig. 8.**
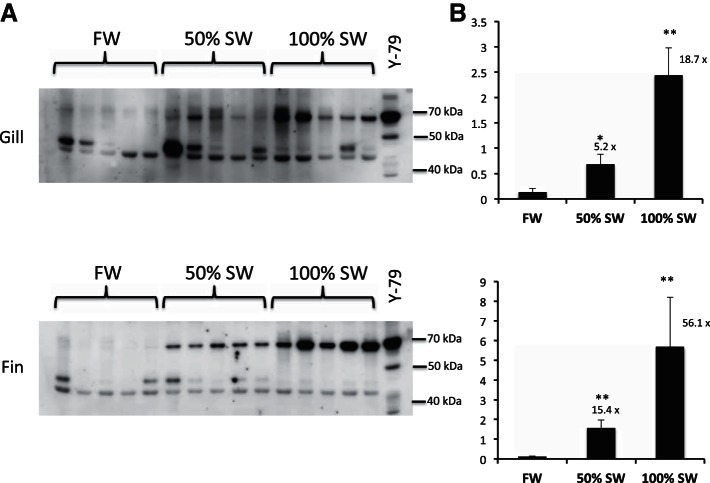
Western blot analysis of MIPS protein expression in tilapia tissues. The blots show the relative abundance of MIPS-immunoreactive proteins in gill and fin supernatant samples (10-μg protein) from FW-, 50% SW-, and 100% SW-acclimated tilapia probed with an anti-hMIPS antibody (Abnova), as described in materials and methods. The blots also include a sample (15-μg protein) from a human Y-79 whole cell extract as a positive control for hMIPS. Blots (*A*) showing immunoreactive proteins were imaged using a Fuji LAS3000 Image Reader and the tilapia 67-kDa protein bands were quantified using AIDA data analysis software (Raytest, Straubenhardt, Germany) and presented as means ± SE (*B*); *n* = 5. Fold-change relative to FW values are indicated above the bars. *P < 0.05, **P < 0.01, compared with FW-acclimated fish.

In contrast to the findings in the eel, SW acclimation in tilapia resulted in marked increases in the expression of mRNAs for both MIPS splice variants in most tissues tested, including gill (6- and 18-fold), kidney (7- and 20-fold), skin (16- and 32-fold), intestine (no change and 11-fold) and fin (no change and 17-fold) for MIPS_(l)_ and MIPS_(s)_, respectively (Fig. 6). In tilapia acclimated to 50% SW, the only significant change in mRNA level was found in the kidney with 2.5- and 2.2-fold increases in MIPS_(l)_ and MIPS_(s)_, respectively. Values recorded for all other tissues in the 50% SW acclimated fish were not statistically different from the FW group. Although expression of the larger splice variant was consistently increased in most tissues from SW-acclimated fish, the fold-increase (1.9-, 5.3-, 6.1-, 6.8-, and 16.1-fold for intestine, fin, gill, kidney, and skin, respectively) was always lower than that recorded for the smaller, more abundant transcript (10.5-, 17.1-, 13.8-, 19.3-, and 32-fold for intestine, fin, gill, kidney, and skin, respectively). Western blot analyses using the Abnova anti-hISYNA1 antiserum revealed similar patterns of protein expression with 11-fold and 54-fold increases, respectively, in the expression of a 67-kDa protein in the gill and fin of SW-acclimated tilapia (Fig. 7). In a second series of experiments, fish acclimated to 50% SW exhibited 5- and 15-fold increases in expression of the 67-kDa protein in gill and fin, respectively (Fig. 8). Although all anti-human MIPS antibodies (Abnova, Abcam, and Santa Cruz) failed to detect a protein of the expected size of either tilapia MIPS transcript (60.6 and 64.5 kDa), all antibodies detected the 67-kDa tilapia protein and a 68-kDa human protein in Western blots (Figs. 7 and 8). The expression of the 67-kDa protein, presumed to be tilapia MIPS, was highly variable in the kidney and skin samples taken from both FW- and SW-acclimated tilapia groups. In the tilapia kidney, all antibodies indicated the presence of two immunoreactive MIPS proteins of ∼67 kDa in most samples (Fig. 7). In all tissues, additional weaker immunoreactive proteins of lower molecular weight were also found (Figs. 7*A* and 8*A*). Because of the high variability in the intensity of the 67-kDa protein, no statistically significant change in expression was found in either kidney or skin. Reprobing the blots with an anti-α-tubulin antibody indicated that the variation was not due to differential protein loading, although the α-tubulin protein levels in supernatant samples were significantly increased by 2.5- and 13-fold in the gill and skin, respectively, following SW-acclimation (results not shown). The reasons for the apparent variability in expression of the presumed MIPS protein in tilapia skin and kidney are unknown.

#### Immunohistochemistry.

**Fig. 9.**
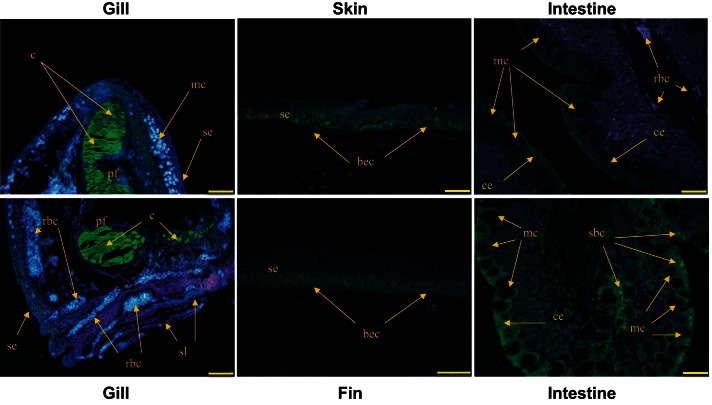
Immunolocalization of MIPS in SW-acclimated tilapia tissues. Specific immunoreactivity (green) was detected in the gill, skin, fin, and intestine. In the gill, immunoreactivity predominated in the chondrocytes of the branchial arch and primary filaments with much lower levels of immunoreactivity distributed throughout all epithelial cells of the primary filaments and secondary lamellae. In skin and fin, immunoreactivity predominated in the basal epithelial cells of the stratified epithelium. In the intestine, immunoreactivity was concentrated just under the apical brush border of the columnar epithelial cells and in certain unknown intestinal regions, throughout the cytoplasm of large mucous cells. Small-bodied cells lying within these densely packed mucous cell regions that are located near the apical surface of the epithelium also exhibited strong immunofluorescence. No specific immunofluorescence was found in sections taken from FW- and SW-acclimated eel nor FW-acclimated tilapia tissues. Cells were counterstained DAPI (blue) to visualize all cell nuclei. bec, basal epithelial cell; c, chondrocyte; ce, columnar epithelium; mc, mucous cell; pf, primary filament; rbc, red blood cell; sbc, small-bodied cells; se, stratified epithelium; sl, secondary lamellae. Yellow bars indicate 75 μm.

Unfortunately, the heterologous anti-MIPS antibodies failed to detect any specific immunoreactivity in tissue sections taken from FW- and SW-acclimated eels, nor did they detect immunoreactivity in tissues taken from FW-acclimated tilapia. However, in SW-acclimated tilapia using the Abnova antibody, specific immunoreactivity predominated in basal epithelial layers of skin, fin, and gill, although in the latter tissue, the highest levels of immunoreactivity were found in the chondrocytes within the branchial arch and the primary filaments (Fig. 9). In the intestine, immunoreactivity was present throughout the columnar epithelial cells and especially close to the apical membrane. In some intestinal regions immunoreactivity was especially abundant throughout the cytosol of large mucous cells and also in small, unknown cell bodies (possibly entero-endocrine cells) interspersed between the columnar epithelial cells (Fig. 9). Specific immunoreactivity was only apparent in tissues taken from SW-acclimated tilapia, although as found with the Western blots, there was considerable heterogeneity in the extent of immunofluorescence in skin and intestine samples isolated from individual fish within the same SW-acclimated group. No specific immunoreactivity was found in tilapia kidney sections taken from any fish group.

## DISCUSSION

Recent studies have indicated that the cyclic alcohol, inositol, functions as an important osmolyte in a number of euryhaline teleosts when they acclimate to SW or hyper-SW environments (10, 15, 24, 25, 39). Inositol accumulates within the cells of internal body tissues, such as the brain, kidney, and liver (10) to compensate for the relatively small increases (5–15%) in plasma osmolality that accompany the transfer of fish to marine environments. In contrast, the apical surfaces of the epithelial cells lining the external body tissues, including the skin, fin, gill, and upper regions of the gastrointestinal tract are faced with much greater osmotic challenges as the external osmolality of <5 mosmol/kg H_2_O found in FW is increased to over 1,000 mosmol/kg H_2_O when fish move to SW. As might be expected, epithelial cells in these tissues must accumulate even higher concentrations of organic osmolytes, including inositol, to maintain their cell volume and, consequently, the integrity of the epithelium. Cellular accumulation of inositol can either be the result of secondary active transport across the plasma membrane by the actions of carriers, such as the sodium myo-inositol transporter (SMIT) and/or the proton myo-inositol transporter (HMIT), as has been reported for various mammalian tissues, including the kidney and brain (6, 12, 28, 41), or can be the result of de novo synthesis from glucose 6-phosphate (36, 43). In the mammalian studies so far reported, neuronal tissues appear to accumulate relatively high concentrations of free inositol (20, 46, 47); however, the brain generally expresses relatively low levels of MIPS mRNA (19), suggesting accumulation via SMIT/HMIT may be more physiologically important than de novo synthesis. However, in the case of euryhaline teleosts, current evidence suggests that the de novo synthesis pathway is a key component of cellular inositol accumulation especially in epithelial tissues exposed to the marine environment (24, 25, 39, 44).

Despite the additional whole genome duplication event in the teleost lineage, only one MIPS gene (*Isyna1*/*Ino1*) has been reported to be present in fish, including the eel and tilapia, suggesting that the duplicated genes were subsequently lost from the teleost genomes during evolution. As expected, the eel and tilapia *Ino1* genes exhibit the high sequence homologies that have been reported to exist for many other species, from yeasts to mammals (17, 21). Both *Xenopus* genes, surprisingly, show much higher-sequence homology to the coelacanth and ghost shark than the lizard, turtle, or birds. The reasons why are currently unknown; however, it was noted that there is almost no synteny conservation around *Ino1* in the amphibian genomes compared with the reptiles, birds, and mammals or the sharks and teleosts (results not shown). Strangely, no *Ino1* has been identified within the zebrafish genome, which questions whether de novo synthesis of inositol is possible in this stenohaline FW species. The sequence currently submitted to the NCBI databank for *O. mossambicus* MIPS has lost 114 nucleotides from exon 7 and includes a nine-nucleotide duplicated insert in exon 3, resulting in a protein of some 35 amino acids less than that of *O. niloticus* (Supplemental Fig. S1). The deletion from exon 7 is strange as it contains one of four highly conserved amino acid motifs found in all other eukaryotes that are considered “core functional structures” for enzyme activity (17, 32, 33). However, the most recently published sequence for *O. mossambicus* MIPS_(l&s)_ (44) by the same group responsible for the current submission (11, 26, 39) suggests that both of these sequence anomalies may have resulted from original sequencing or cloning errors.

In both *O. niloticus* (this study) and *O. mossambicus* (39), two MIPS splice variants exist where the inclusion of an 87-nucleotide insert from the intron between exons 5 and 6 results in the additional expression of a longer transcript, MIPS_(l)_. The additional 29 amino acids of the larger splice variant includes a short region that contains a number of potential phosphorylation sites within the protein (i.e.. . . . KVSDSPRYSSVY. . . . ), which again suggests that the activity of this form of the enzyme may be differentially regulated by specific kinases and phosphatases. Both tilapia splice variants appear to undergo NH_2_-terminal acetylation (39); however, the functional significance of this protein modification is not known. Although there is the potential for a similar splice variant in the eel, the expression of this larger transcript could not be detected in any tissue from FW- or SW-acclimated fish. The predominant tilapia MIPS_(s)_ splice variant and the single eel MIPS enzyme also harbor a number of additional potential phosphorylation sites (Supplemental Fig. S1) and, indeed, the enzymes extracted from rat brain and testes have been shown to contain similar phosphorylation sites close to the COOH-terminus (37), and activities of both the yeast and human enzymes have been reported to be inhibited by multiple phosphorylations (7). In almost all *O. niloticus* tissues tested, the shorter splice variant, MIPS_(s)_, is expressed at much higher levels (>10-fold) than the longer splice variant, MIPS_(l)_, although levels of the larger splice variant are slightly higher in the oesophagus and intestine, where the ratios of the two transcripts (7- and 3-fold) are more similar to that reported in the gill of *O. mossambicus* (39). The major exceptions to this are in the oocytes and white skeletal muscle where the expression of MIPS_(l)_ mRNA was ∼20-fold higher than that of MIPS_(s)_. The difference in abundance of MIPS transcripts seen in oocytes and skeletal muscle suggest that MIPS may be differentially regulated and inositol may have additional functions within these tilapia tissues.

With the exception of small (2.5-fold) increases in expression in the kidney, general tissue levels of MIPS mRNA in the eel were not affected by SW acclimation. In contrast, SW acclimation induced substantial increases in mRNA abundance of one or both tilapia MIPS transcripts in all tissues tested. Although both tilapia MIPS transcripts appeared to exhibit some degree of salinity-sensitive expression, SW acclimation was accompanied by greater increases in the expression of the more abundant MIPS_(s)_ splice variant than the larger MIPS_(l)_ splice variant. This is in general agreement with the increases in mRNA expression reported for both MIPS splice variants in the brain and gill of *O. mossambicus* when exposed either acutely or gradually (up to 2 wk) to SW or to hyper-saline environments of 70 and 90 ppt (15, 39).

Although Western blots failed to detect proteins with the expected molecular weights of tilapia or eel MIPS (60.4–64.5 kDa), in both species, a number of other tissue-specific immunoreactive proteins of varying, but similar molecular weights, were observed. In the eel, proteins of ∼67, 45, and 40 kDa were identified, which cross-reacted with the heterologous antibodies. In the tilapia, a protein of 67 kDa predominated in all tissues, although a number of less abundant/immunoreactive proteins ranging in molecular weight between 40 and 60 kDa were also seen. Western blots also resulted in the identification of a 68-kDa immunoreactive protein in a human cell extract, presumed to be the 61-kDa human MIPS protein. Endogenously expressed MIPS proteins found in other species have often been reported to migrate at molecular weights 7–8 kDa higher than that expected from their known amino acid sequences (30, 42). In addition, spliced variants of MIPS mRNA are known to result in the expression of differently sized functional proteins in plants (22), insects (2), and mammals (37, 42). Three differentially spliced NH_2_-terminal variants have been reported in the database for the human MIPS gene, which would result in putative proteins with molecular weights of 61.1, 55.1, and 47.2 kDa (see Table 2). These observations, together with the consistency of the appearance of the 67-kDa protein in blots from most tissues in both species and with the general changes in expression agreeing with the species-specific mRNA levels found, suggest that these proteins may, indeed, be different forms of MIPS.

In the eel gill, fin, kidney, and intestine, SW acclimation failed to have any significant effect on any of the proteins exhibiting cross-reactivity with the anti-MIPS antibodies, which is in general agreement with the lack of change in MIPS mRNA expression. The true identities of the lower-molecular-weight proteins await future characterization; however, the intriguing possibility remains that they are all truncated forms of eel MIPS. The increases in mRNA expression associated with SW acclimation in *O. niloticus* were accompanied by similar increases in the expression of the 67-kDa protein, presumed to be full-length MIPS, in gill and fin. Whether this protein is related to MIPS_(l)_ or MIPS_(s)_ remains to be determined. Although SW acclimation resulted in consistent and significant upregulation of both MIPS_(l)_ and MIPS_(s)_ mRNAs in kidney and skin, the levels of expression of the 67-kDa protein were highly variable in these tissues with some SW-acclimated fish exhibiting expression levels similar to or even below that of FW-acclimated fish. Because of the variability found in the levels of expression of all immunoreactive proteins found in tilapia kidney and skin, no significant upregulation of any protein was seen following SW acclimation. The large increases in expression of the presumed 67-kDa MIPS protein found in the gill of SW-acclimated *O. niloticus* is not paralleled by the results found in *O. mossambicus* gill, where much lower (2–3-fold) increases in protein abundance were found after 2 days and 13 days of stepwise acclimation to 34 ppt and 90 ppt salinity, respectively (39). Strangely *O. mossambicus* acclimated in a stepwise manner to 70 ppt salinity over 10 days and exhibited no significant increases in gill MIPS protein. The relatively subdued upregulation in MIPS protein in *O. mossambicus* gill was attributed to a generalized suppression of translation due to the salinity stress (39).

Despite the lack of any direct evidence, it is highly likely that posttranslational modifications, including protein acetylation and phosphorylation, may affect MIPS enzyme abundance, location, and/or activity. In addition, a MIPS homolog expressed in the seaweed *Gracilaria lemaneiformis* has been shown to be a calmodulin-binding protein with the potential to be regulated by cytosolic calcium (49). The putative 25 amino acid calmodulin-binding domain exhibits 68% and 88% identity and similarity, respectively, with the aligned sequence in tilapia. The calmodulin-binding domain is found toward the COOH-terminal end of the Rossmann fold (responsible for binding NAD^+^) and includes a highly conserved 9 amino acid motif (. . . V/LWTANTER/CF/Y. . .) with unknown function that is found in all eukaryotic MIPS proteins (Refs. 17 and 32; Supplemental Fig. S1). Although the effects of posttranslational modifications and second messenger regulation of teleost IMPA and MIPS have not been studied, the activity of the recombinant tilapia enzymes have been shown to be markedly affected by ionic strength and pH (44). Increasing the pH results in enzymatic activities of both enzymes increasing by 20- to 100-fold over the pH range 7–9. Likewise, changes in ionic strength and sodium/potassium ratios have profound effects on the kinetics of both enzymes and the specificity of IMPA for different IP isomers (44).

MIPS is generally considered to be the main regulator and rate-limiting enzyme in the de novo synthesis of inositol in most species, from plants (27, 48) to the higher vertebrates and mammals (1, 7, 23). However, the expression data found in this and previous studies (24, 25), and in recent reports from the Kültz group (15, 39, 44), indicate that regulation of IMPA expression may be just as, if not more, important in the regulation of inositol production in euryhaline teleosts, such as the eel and tilapia. In addition, homologues of the inositol transporters SMIT and HMIT are also present in teleost genomes; however, the role played by these proteins in the cellular accumulation of inositol in different tissues in the eel and tilapia await future investigation.

### Perspectives and Significance

Apart from its ubiquitous presence in all tissues and the predominance of expression in the brain, the relative MIPS mRNA tissue abundance profile was unique to each species. The expression of the larger MIPS splice variant appears to be confined to tilapia, and its specific functional roles (especially in skeletal muscle and oocytes) await further investigation. With the exception of small increases in mRNA abundance in the kidney, SW acclimation had no effect on the expression of MIPS in *A. anguilla*. This was in contrast to *O. niloticus* in which significant increases in mRNA and in some cases protein expression were found in tissues from SW-acclimated fish. Because of differences in the experimental setups, it is difficult to make direct comparisons between the results found here with *O. niloticus* and those reported previously for the more euryhaline *O. mossambicus* species. However, it would appear that in the less euryhaline *O. niloticus* species, substantial increases in expression of mRNA and protein for MIPS are required for successful acclimation to SW. Given the more subdued upregulation of MIPS expression in response to salinity in both *O. mossambicus* and *A. anguilla*, the requirement for inositol biosynthesis for successful osmoregulation in SW may be less acute in these species that are known to exhibit more robust acclimation to SW or hyper-saline environments. This would be consistent with the finding that salinity-induced increases in tissue contents of inositol and other osmolytes are much greater in the tilapia than the eel (24). However, in both species, previous studies have shown that SW acclimation results in a marked increase in the expression of the IMPA1.1 isoform. This suggests that, unlike that reported in mammals, it is the dephosphorylation of inositol phosphate that is the most highly regulated and rate-limiting step in inositol biosynthesis in teleosts rather than the production of inositol phosphate by MIPS.

## GRANTS

The work was funded by a research grant awarded to G. Cramb and N. Hazon by the Natural Environment Research Council (NE/J010081/1).

## DISCLOSURES

No conflicts of interest, financial or otherwise, are declared by the authors.

## AUTHOR CONTRIBUTIONS

S.K., N.H., and G.C. conception and design of research; S.K. and G.C. performed experiments; S.K. and G.C. analyzed data; S.K. and G.C. interpreted results of experiments; S.K., N.H., and G.C. edited and revised manuscript; S.K., N.H., and G.C. approved final version of manuscript; G.C. prepared figures; G.C. drafted manuscript.

## Supplementary Material

Supplemental Figure S1

Supplemental Table S1
