# Supplementary material for: Myo-inositol phosphate synthase expression in the European eel (Anguilla anguilla) and Nile tilapia (Oreochromis niloticus): effect of seawater acclimation
Source: Am J Physiol Regul Integr Comp Physiol. 2016 Jun 1;311(2):R287–98. doi: 10.1152/ajpregu.00056.2016 (PMC5008666; doi:10.1152/ajpregu.00056.2016)
Supplement: Supplemental Figure S1 [file Supplemental_Figure_S1.pdf]

```
Caenorhabditis_elegans             MSSAQVNGISKRLIVESPNVKLEDGVLESRFTYRKNHFEHRA-DGLHVTPKEHDYSFKTV   59
Drosophila_melanogaster            ----MKPTNNSTLEVISPKVQVDDEFITTDYDYQTSHVKRTADGQLQVHPQTTSLKIRTG   56
Culex_quinquefasciatus             --------MASSLNVESPNVHYSDKHIEVDYQYQTTSVLAEGPAGYTVRPETTKLSIRTD   52
Aedes_aegypti                      --------MASALNVVSPNVHYSDKYIEVDYQYQTTSVLNEGPSGYTVRPETTELSIRTE   52
Petromyzon_marinus                 --------MAEMFQVQSPNVRYTEEHIEAVYQYQTSHVARHN-GRLIVSPQTKEYEFRTR   51
Apis_florea                        --------MALKIRVQSPKVKYTEDCIEAQYEYQTTNVIEDD-TKYTVTPVSTKLLIRTQ   51
Strongylocentrotus_purpuratus      ---------MTSFTVESPNVRYTHQHIESDYAYQTTKVEKNG-QNIKVIPVDVQMKFRTE   50
Mus_musculus                       ------MEPAAEILVDSPDVVYSPETIEARYEYRTTRVSREG-GVLRVQPRATRFTFRTA   53
Saimiri_boliviensis                --------------------------------------------------------MR--   2
Homo_sapiens                       ------MEAAAQFFVESPDVVYGPEAIEAQYEYRTTRVSREG-GVLKVHPTSTRFTFRTA   53
Bos_taurus                         ------MEAATEFVVESPDVVYSPETIEAQYEYRTTSVSREG-GVLKVHPTSTRFTFRTA   53
Canis_lupus                        ------MEASAEFVVESPDVVYGPDAIEAQYEYRTTCVSRED-GVLKVYPTSTRFTFRTA   53
Salmo_salar                        --------MTENIRINNPNVKYTDTHIEAQYFYHTTAVRRDG-DTLTVTPSVSELEFRTE   51
Gadus_morhua                       -----------------------------------------------VSPGTHEMTFRTE   13
Takifugu_rubripes                  --------MSVNVRINSPNVQYTDAHITARYSYQTTSVHRDG-NNITASPCATEMTFRTE   51
Tetraodon_nigroviridis             --------MSVNIHINSPNVQYTDAHITARYSYQTTSVHAEE-NNITVTPCSTEVTFRTE   51
Xiphophorus_maculatus              --------MSASIHVNSPNVKYTDTHIEAHYSYQTTSVRQEG-NRVTVTPRSTEMTFRTE   51
Oryzias_latipes                    --------MSVNIHINSPNVKYTDTHIFAKYSYESTSVQQDG-SNITVTPRSTEMTFRTE   51
Gasterosteus_aculeatus             --------MSANVHINSSNVKYTDTHIVSQYSYQTTSVQRDG-NKVTVTPRTTEMTFRTE   51
Oreochromis_mossambicus_v1         --------MSVNVHINSPNVKYTDSHIEAQYSYQTTSVHRDG-NKVTVTPRTTEMTIRTE   51
Oreochromis_mossambicus_v2         --------MSVNVHINSPNVKYTDSHIEAQYSYQTTSVHRDG-NKVTVTPRTTEMTIRTE   51
Pundamilia_nyererei                --------MSVNVQVNSPNVKYTDSHIEAQYSYQTSSVHRDG-NKVTVTPRTTEMTIRTE   51
Pseudotropheus_zebra               --------MSVNVQVNSPNVKYTDSHIEAQYSYQTSSVHRDG-NKVTVTPRTTEMTIRTE   51
Oreochromis_niloticus_v1           --------MSVNVHINSPNVKYTDSHIEAQYSYQTTSVHRDG-NKVTVTPRTTEMTIRTE   51
Oreochromis_niloticus_v2           --------MSVNVHINSPNVKYTDSHIEAQYSYQTTSVHRDG-NKVTVTPRTTEMTIRTE   51
Anolis_carolinensis                --------MAAPFVVESPDVRYTPDVIEAQYDYQAVQVRQED-GITKVTPSSTRLLFRTE   51
Anguilla_anguilla_v1               --------MAEPVRINSPDVRYTAKHIEAQYDYHTTTVSCDD-GKYTVTPLTTRYTFRTE   51
Sarcophilus_harrisii               --------MEETFVVESPDVTYGPDVIQAHYTYQTTRVSQDG-GSLKVHPISTRFTFRTE   51
Chrysemys_picta_bellii             --------MAETFLVESPDVTYSKDYIEAKYTYSTVHVSRED-GVTKVKPQSTEFTFRTE   51
Ficedula_albicollis                --------MAEPFLVESPNVTYSKDFIEAKYTYSTVHVCKEN-GVTKVRPCSTRFTFRTG   51
Gallus_gallus                      --------MAEPFVVESPDVAYSKDFIEAQYTYSTAHVCREG-GVTKVRPCSTRFTFRTA   51
Cyprinus_carpio                    --------MPEKVRINSADVKYTEKHIESRYCYNTASVRRDG-DTFTVTPSSTEFIFRTE   51
Callorhinchus_milii                --------MADMFFVESPYVTYTKDSIEAQYSYQTTEVFQEN-GKVKVKPCSTKFTFCTE   51
Latimeria_chalumnae                -----------------------------------------------VKPCSTKFTFRTQ   13
Xenopus_laevis                     --------MAEKFKVESPHVRYLKDVIEADYNYDTTQVYEEK-GVTKVKPCSTKFTFHTE   51
Xenopus_tropicalis                 --------MAEKFKVESPHVRYLKDVIEADYNYDTTQVYEEK-GVTKVKPCSTKFTFHTE   51
                                                                                           :

Caenorhabditis_elegans             L---KPRKTGLLLVGLGGNNGSTAVGSIFANQYAMTWRTKEGHSQANYFGSVTQTATVHL   116
Drosophila_melanogaster            R---HVPKLGVMLVGWGGNNGSTLTAALEANRRQLKWRKRTGVQEANWYGSITQASTVFI   113
Culex_quinquefasciatus             R---DVPKMGLMLAGWGGNNGSTLTAALEANKRGLEWRTRTGMQKANWFGSITQASTVLL   109
Aedes_aegypti                      R---QIPRLGLMLVGWGGNNGSTITAALEANKRGLEWRTRTGVQKANWFGSITQASTVLL   109
Petromyzon_marinus                 R---HVPRLGVMLVGWGGNNGSTVTAATIANRLGLSWNTKTGVKHANYFGSVFQSSTVSL   108
Apis_florea                        L---KVPKLGLMLVGWGGNNGTTITAALLANKLKLTWDTKNGIQKANWYGSLIQASTIRL   108
Strongylocentrotus_purpuratus      R---KVPRLGVMLVGWGGNNGSTVTGAVIANQMKMSWRTKEGTRHADYYGSLTQASTLNL   107
Mus_musculus                       R---QVPRLGVMLVGWGGNNGSTLTAAVLANRLRLTWPTRTGRKEANYYGSLTQAGTVNL   110
Saimiri_boliviensis                ------------------------SPRPLVTTPTRHLALSAAPQEANYYGSLTQVGTVSL   38
Homo_sapiens                       R---QVPRLGVMLVGWGGNNGSTLTAAVLANRLRLSWPTRSGRKEANYYGSLTQAGTVSL   110
Bos_taurus                         R---QVPRLGVMLVGWGGNNGSTLTAAVLANRLRLSWPTRTGRKEANYYGSLTQAGTVSL   110
Canis_lupus                        R---QVPRLGVMLVGWGGNNGSTLTAAVLANRLRLSWPTRTGRKEANYYGSLTQAGTVSL   110
Salmo_salar                        R---RVPRLGVMLVGWGGNNGTTVTAAVLANRLGLTWRTKTGEQKANYYGSLFQSSTVCL   108
Gadus_morhua                       R---TVPRVGVMLVGWGGNNGSTVTAAVLANRMGLSWTTKTGEKRANYFGSLFQSSTVSL   70
Takifugu_rubripes                  R---RVPRLGVMLVGWGGNNGTTVTAAVLANSLGLTWSTKTGQKKANYFGSFLQASTVCL   108
Tetraodon_nigroviridis             R---RVPRLGVMLVGWGGNNGTTVTAAVLANSLGLAWRTKTGLKKANYFGSLLQASTVCL   108
Xiphophorus_maculatus              R---RVPRLGVMLVGWGGNNGTTVTAAVLANKLGLTWRTKNGEKKANYYGSLLQASTVCL   108
Oryzias_latipes                    R---RVPRLGVMLVGWGGNNGTTVTAAVLANKLGLTWRTKNGEMKANYYGSLLQSSTVCL   108
Gasterosteus_aculeatus             R---RVPRLGVMLVGWGGNNGTTVTAAVLANKLGLTWRTNTGVKKANYYGSLLQASTVCL   108
Oreochromis_mossambicus_v1         RRVPRVPRLGVMLVGWGGNNGTTVTAAVLANKMGLTWKTKNGVKKANYFGSLLQSSTVCL   111
Oreochromis_mossambicus_v2         RRVPRVPRLGVMLVGWGGNNGTTVTAAVLANKMGLTWKTKNGVKKANYFGSLLQSSTVCL   111
Pundamilia_nyererei                R---RVPRLGVMLVGWGGNNGTTVTAAVLANKLGLTWKTKNGVKKANYFGSLLQSSTVCL   108
Pseudotropheus_zebra               R---RVPRLGVMLVGWGGNNGTTVTAAVLANKLGLTWKTKNGVKKANYFGSLLQSSTVCL   108
Oreochromis_niloticus_v1           R---RVPRLGVMLVGWGGNNGTTVTAAVLANKMGLTWKTKNGVKKANYFGSLLQSSTVCL   108
Oreochromis_niloticus_v2           R---RVPRLGVMLVGWGGNNGTTVTAAVLANKMGLTWKTKNGVKKANYFGSLLQSSTVCL   108
Anolis_carolinensis                R---RVPKLGVMLVGWGGNNGTTVTAAVLANKLGLTWMTKTGPKRANYYGSLLQASTVCL   108
Anguilla_anguilla_v1               R---TVPRLGVMLVGWGGNNGTTVTAAVLANKLGLSWRTKEGLKKANYFGSLLQASTVSL   108
Sarcophilus_harrisii               R---KVPRLGVMLVGWGGNNGSTVTAAVLANKLKLSWMTKTGRKEANYYGSLMQAATVSL   108
Chrysemys_picta_bellii             R---QVPRLGVMLVGWGGNNGTTVTAAVLANRLGLSWMTKTGRKYANYYGSLLQASTVCL   108
Ficedula_albicollis                R---QVPRLGVMLVGWGGNNGTTVTAAVLANKLGLSWMTKTGRKKANYYGSLLQASTVCL   108
Gallus_gallus                      R---HVPRLGLMLVGWGGNNGTTVTAAVLANRLGLSWMTKTGRKKANYYGSLLQASTVCL   108
Cyprinus_carpio                    R---KVPRLGVMLVGWGGNNGTTVTAAVLANKLGLTWKTKTGVKKANYFGSLLESSTVCL   108
Callorhinchus_milii                R---KVPKLGVMLIGWGGNNGTTVTAAVLANKLGLSWMTKTGSKDANYYGSLFQASTVCL   108
Latimeria_chalumnae                R---KVPRLGVMLVGWGGNNGTTVTAAILANKMGLSWNTKTGIKHANYYGSFLQSSTACL   70
Xenopus_laevis                     R---KVPKLGVMLVGWGGNNGTTVTAAVLANRLGLSWMTKTGKKVANYYGSLFQSSTACL   108
Xenopus_tropicalis                 R---KVPKLGVMLVGWGGNNGTTVTAAVIANQLGLSWMTKTGKKVANYYGSLFQSSTVCL   108
                                                                  ..          .   *:::**. : .*  :

Caenorhabditis_elegans             GYDSATQNQIFVPFKDIVPILSPNDLIISGWDISDSNLYEAMGRAKVFEPELQEKLRPFM   176
Drosophila_melanogaster            GSDEDG-GDVYVPMKELLPMVEPDNIIVDGWDISGLHLGDAMRRAEVLDVALQDQIYDQL   172
Culex_quinquefasciatus             GTDANG-HDVHIPMNKLVPMVNPDDIVVDGWDISSMNIGDAMVRAKVLEVPLQDQVYKKL   168
Aedes_aegypti                      GTDAKG-QDVHIPMNKLVPMVNPDDIVVDGWDISSMNIGEAMIRAGVLEVGLQDQVHKKL   168
Petromyzon_marinus                 GSGPQG--DVFIPFRDLLPMVHPNDIVFDGWDISSLNLAEAMRRAEVLDWGLQEQLRPHM   166
Apis_florea                        GKGNKE--DIYVPMSWMLPIVNPDDIQIDGWDISDMNLADAMQRAKVLDINLQKQLVEYM   166
```

```
Strongylocentrotus_purpuratus      GSGPAG--DLNIPFKDILPMVDPNDIVFDGWDISSFNLAEAMERAQVFDWSLQEQLRPHM   165
Mus_musculus                       GLDENG-REVFVPFSALLPMVAPNDLVFDGWDISSLNLAEAMRRAQVLDCGLQEQLWPHM   169
Saimiri_boliviensis                GLDAEG-QEVFVPFSALLPMVAPNDLVFDGWDISSLNLAEAMRRAKVLDWELQEQLWPHM   97
Homo_sapiens                       GLDAEG-QEVFVPFSAVLPMVAPNDLVFDGWDISSLNLAEAMRRAKVLDWGLQEQLWPHM   169
Bos_taurus                         GLDAEG-KEVFVPFSSLLPMVAPDDLVFDGWDISSLNLAEAMRRAQVLDWGLQEQLWPHM   169
Canis_lupus                        GLDAEG-QEVFVPFSALLPMVAPDDLVFDGWDISSLNLAEAMRRAQVLDWGLQEQLWPHL   169
Salmo_salar                        GAGPDG-KEVNIPFRDLLPMVHPNDIVFDGWDISSMDLGSAMERAEVLDWSLQEKLRPHM   167
Gadus_morhua                       GMAPEG--EVHVPFRDLLPMVHPDDIIFDGWDISSMDLGRAMERAQVLDWSLQEELRPHM   128
Takifugu_rubripes                  GSGLEG--DVNIPFRDLLPMVHPNDIVFDGWDISSMDLGCAMERAQVFDWSLQEQLRPHL   166
Tetraodon_nigroviridis             GSGPEG--DVNIPFRDLLPMVHPNDIVFDGWDISSMDLGRAMERAQVFDWSLQEQLRPYM   166
Xiphophorus_maculatus              GSGLEG--EVNVPFRDLLPMVHPNDIVFDGWDISSLDLGRAMERAQALDWSLQEQLRPYM   166
Oryzias_latipes                    GSGLEG--EVNVPFRDVLPMVHPNNIVFDGWDISSMDLGRAMERAKVLDWSLQEQLRPYM   166
Gasterosteus_aculeatus             GTGVEG--EVNVPICDLLPMVHPNDIVFDGWDISSMDLGRAMERAQVLDWSLQEQLRPHL   166
Oreochromis_mossambicus_v1         GSGLEG--EVNVPFRDLLPMVHPNDIVFDGWDISSLDLGSAMERAQVLDWSLQEQLRPYM   169
Oreochromis_mossambicus_v2         GSGLEG--EVNVPFRDLLPMVHPNDIVFDGWDISSLDLGSAMERAQVLDWSLQEQLRPYM   169
Pundamilia_nyererei                GSGLEG--EVNVPFRDLLPMVHPNDIVFDGWDISSLDLGSAMERAQVLDWSLQEQLQPHM   166
Pseudotropheus_zebra               GSGLEG--EVNVPFRDLLPMVHPNDIVFDGWDISSLDLGSAMERAQVLDWSLQEQLQPHM   166
Oreochromis_niloticus_v1           GSGLEG--EVNVPFRDLLPMVHPNDIVFDGWDISSLDLGSAMERAQVLDWSLQEQLRPYM   166
Oreochromis_niloticus_v2           GSGLEG--EVNVPFRDLLPMVHPNDIVFDGWDISSLDLGSAMERAQVLDWSLQEQLRPYM   166
Anolis_carolinensis                GNGPGG--EVYVPFRDLLPMVHPDDIVFDGWDISGMNLAEAMRRAQVLDWPLQEQLRPHM   166
Anguilla_anguilla_v1               GTGPEG--EVYVPFRDLLPMVHPNDIIFDGWDISSMDLGQGMERAKVLDWALQEQLRPYM   166
Sarcophilus_harrisii               GSDDHG-QNVHVPFRALLPMVSPDDIVFDGWDISSLNLAEAMKRAQVLDWALQEQLWPHM   167
Chrysemys_picta_bellii             GTGPTG--DVYVPFRDLLPMVHPNDIVFDGWDVSSLNLAEAMRRAKVLDWQLQEQLWPHM   166
Ficedula_albicollis                GTGPSG--DVYVPFRDLLPMVHPNDIVFDGWDISSLNLAEAMKRAEVLEWPLQEQLWPHM   166
Gallus_gallus                      GAGPTG--DVYVPFRDLLPMVHPNDIVFDGWDISSLNLAEAMRRAEVLDWALQEQLWPHM   166
Cyprinus_carpio                    GSGPDG--EVYTPFRDLLPMVHPNDIVFDGWDISSLDLGRAMERAQVLDWSLQEKLRPHM   166
Callorhinchus_milii                GTGPAG--DYYVPFKDLLPMVNPNDIIFDGWDISSMNLADAMQRAKVLDWELQEQLRPHM   166
Latimeria_chalumnae                GSGPNG--DVYVPFRDLLPMVHPNDIVFDGWDISSLNMAQAMERAQVLDWQLQEQLRPHM   128
Xenopus_laevis                     GSGPAG--DVFVPFRDLLPMVHPNDIVFDGWDISSLNLADAMFRAEVLDWQIQEQLRPYM   166
Xenopus_tropicalis                 GTGPAG--EVFVPFRDLLPMVNPNDIVFDGWDISSLNLADAMFRAEVLDWQLQEQLRPYM   166
                                   *       :   *:  ::*:: *::: ..***:*  .:   .* ** .::  :*.::   :

Caenorhabditis_elegans             EPIVPLPSIYYPDFIASNQGDRANNVIPGDNKL---------------------------   209
Drosophila_melanogaster            AQLRPRPSIYDPDFIAANQSDRADNVIRGTRLE---------------------------   205
Culex_quinquefasciatus             SQLKPRPSIYDPDFIAANQADRADNTIPGTRYQ---------------------------   201
Aedes_aegypti                      SLMKPRPSIYDPDFIAANQADRADNTIPGTRYE---------------------------   201
Petromyzon_marinus                 EKLRPRASIYVPEFIAANQEDRANNLIPGTKAE---------------------------   199
Apis_florea                        IHMKPRKSIYYSDFIAANQEKRANNVISGTKFE---------------------------   199
Strongylocentrotus_purpuratus      ATLKPRPSVYFPDFIAANQEDRADNVLTGTKSE---------------------------   198
Mus_musculus                       ESLRPRPSVYIPEFIAANQTARADNLIPGTRAQ---------------------------   202
Saimiri_boliviensis                EALRPRPSVYIPEFIAANQSTRADNLIPGSRAQ---------------------------   130
Homo_sapiens                       EALRPRPSVYIPEFIAANQSARADNLIPGSRAQ---------------------------   202
Bos_taurus                         EAMRPRPSVYIPEFIAANQSARADNVIPGTRAQ---------------------------   202
Canis_lupus                        EALRPRPSVYIPEFIAANQSVRADNLILCTRAQ---------------------------   202
Salmo_salar                        SHLRPRASIYIPEFIAANQAGRADNVLKGTMAE---------------------------   200
Gadus_morhua                       SSLKPRPSIFIPEFIAANQESRADNVLSGTIAE---------------------------   161
Takifugu_rubripes                  SLMKPRPSIYIPEFIAANQESRADNVLTGTLAD---------------------------   199
Tetraodon_nigroviridis             SRMKPRPSIYIPEFIAANQESRADNVLTGTMAE---------------------------   199
Xiphophorus_maculatus              SSLKPRPSIYIPEFIAANQESRADNVLTGTMVE---------------------------   199
Oryzias_latipes                    SCLKPRPSIYIPEFIAANQESRADNVLTGTLTE---------------------------   199
Gasterosteus_aculeatus             SQMKPRPSIYIPDFIAANQESRADNVLTGSIAE---------------------------   199
Oreochromis_mossambicus_v1         SCLKPRPSIYIPEFIAANQESRADNVLTGTMAE---------------------------   202
Oreochromis_mossambicus_v2         SCLKPRPSIYIPEFIAANQESRADNVLTGTMAEQVIKVSDSPFYSSVYFFSLAICLKECM   229
Pundamilia_nyererei                SCLKPRPSIYIPEFIAANQESRADNVLTGTMAE---------------------------   199
Pseudotropheus_zebra               SCLKPRPSIYIPEFIAANQESRADNVLTGTMAE---------------------------   199
Oreochromis_niloticus_v1           SSLKPRPSIYIPEFIAANQESRADNVLTGTMAE---------------------------   199
Oreochromis_niloticus_v2           SSLKPRPSIYIPEFIAANQESRADNVLTGTMAEQVIKVSDSPFYSSVYFFSLAICLKEYM   226
Anolis_carolinensis                EGMRPRPSIYIPEFIAANQQERADNVLTGTKAE---------------------------   199
Anguilla_anguilla_v1               AKLKPRPSVYYPEFIAANQKDRADNLITGTKAE---------------------------   199
Sarcophilus_harrisii               EQLRPRPSVYIPKFIAANQESRADNLISGSRAQ---------------------------   200
Chrysemys_picta_bellii             VTMKPRPSIYIPGFVADNQEDRADHVLHGTKAE---------------------------   199
Ficedula_albicollis                EKMKPRPSIYIPEFIAANQEERADNVLRGSMAE---------------------------   199
Gallus_gallus                      EQMKPRPSIYIPEFIAANQEERADNVLRGSKAE---------------------------   199
Cyprinus_carpio                    SQIKPRPSVYIPEFIAANQEQRADNLIRGSKVE---------------------------   199
Callorhinchus_milii                RNLQPRPSIYIPAFIAANQKDRADNVLQGSKFE---------------------------   199
Latimeria_chalumnae                EQMKPRPSIYIPEFIAANQEGRADHVIDGTKAE---------------------------   161
Xenopus_laevis                     EKMKPRPSIYIPDFIAANQEDRADHTIHGTKAE---------------------------   199
Xenopus_tropicalis                 EKMKPRPSIYIPEFIAANQEDRANHIIHGTKAE---------------------------   199
                                     : *   *::    *:* **   **:. :

Caenorhabditis_elegans             -EHLEHIRADIRKFKQEHELECVIVLWTANTERYTDVRQGLNATADEIMESIRVNEDEVS   268
Drosophila_melanogaster            --QYEQIRKDIRDFRERSGVDSVIVLWTANTERFADVQPGLNTTSQELIASLEANHSEVS   263
Culex_quinquefasciatus             --QYQQIVKDIQDFKKSSGVEKVVVLWTANTERFSEVQKGLNTTMAELEKSLKENKSEIS   259
Aedes_aegypti                      --QYQQIVKDIREFKRTSGVDKVVVLWTANTERFADVKKGLNTTMAELEKSLKEN-SEIS   258
Petromyzon_marinus                 --QLEQLRRDIRDFKASSGVEKVIVMWTANTERFCEVAVGLNDTSDSLMKSIARNESEVS   257
Apis_florea                        --QLEQIRKDIAEFKNSNKLDQVIILWTANTERFSEIIPGVNDTAENLLNAIKKSHSEVS   257
Strongylocentrotus_purpuratus      --LVEKIRADIREFKQ--KVDKVIVLWTANTERFSDVAEGLNTTEHALLMSIETNANEVS   254
Mus_musculus                       --QLEQIRKDIRDFRSSAGLDKVIVLWTANTERFCEVVPGRNDTAENLLHTIQLG-LEVS   259
Saimiri_boliviensis                --QLEQIRKDIRDFRSSAGLDKVIVLWTANTERFCEVVPGLNDTAENLLRTIELG-REVS   187
Homo_sapiens                       --QLEQIRRDIRDFRSSAGLDKVIVLWTANTERFCEVIPGLNDTAENLLRTIELG-LEVS   259
Bos_taurus                         --QLEQIRRDIRDFRFSAGLDKVIVLWTANTERFCEVIPGLNDTAENLLRTIQLG-LEVS   259
Canis_lupus                        --QLEQIRRDIRDFRSSAGLDKVIVLWTANTERFCEVIPGLNDTAENLLRTIQLG-LEVS   259
Salmo_salar                        --QVEQIRADIQDFRHSSGVDKVIVLWTANTERFCDLTAGVNDTAKNLLAAIQSG-GEVS   257
Gadus_morhua                       --QVEQIRADIRDFWQSSGVDKVIVLWTANTERFCDLTPGVNDTAENLLATIQGG--EVS   217
```

```
Takifugu_rubripes                   --QVQQIRADIRDFQQSSGVDKVIVLWTANTERFTDIVPGVNDSADGLLAAIQAG-AEVS   256
Tetraodon_nigroviridis              --QVEQIRADIRDFQQSSGVDKVIILWTANTERFTDIIPGVNDSADSLLAAIQAG-EGVS   256
Xiphophorus_maculatus               --QMEQIRADIRDFRQSSGVDKVIVLWTANTERFCDIVPGVSDSAKNLLAGIQAGAAEIS   257
Oryzias_latipes                     --QVEQIRADIRDFRQSSGVDKVIVLWTANTERFCDIVPGINDTAKNLLAAIQAGA-EVS   256
Gasterosteus_aculeatus              --QVEQVRADIRDFRQSSGVDKVIVLWTANTERFCDVIPGVNDKAKNLLAAIQAG-AEVS   256
Oreochromis_mossambicus_v1          --QMERIRADIRDFRQASGVDKVIVLWTANTERFCDIIPGVNDAAKNLLAAIQAG-----   255
Oreochromis_mossambicus_v2          IFQMERIRADIRDFRQASGVDKVIVLWTANTERFCDIIPGVNDAAKNLLAAIQAG-----   284
Pundamilia_nyererei                 --QMERITADIRDFRQASGVDKVIVLWTANTERFCDIIPGVNDSAKNLLAAIQAGAAEVS   257
Pseudotropheus_zebra                --QMERITADIRDFRQASGVDKVIVLWTANTERFCDIIPGVNDSAKNLLAAIQAGAAEVS   257
Oreochromis_niloticus_v1            --QMERIRADIRDFRQASGVDKVIVLWTANTERFCDIIPGVNDSAKNLLAAIQAGAAEVS   257
Oreochromis_niloticus_v2            IFQMERIRADIRDFRQASGVDKVIVLWTANTERFCDIIPGVNDSAKNLLAAIQAGAAEVS   286
Anolis_carolinensis                 --QVAQIRRDIRDFRNASGVDKVIVLWTANTERFCEVQTGLNDTAENLLRAIESG-LEVS   256
Anguilla_anguilla_v1                --QLDQIRRDIQDFKQKGGVDKVIVLWTANTERFCDIIPGVNDTAKNLLAAIQTG-AEVS   256
Sarcophilus_harrisii                --QLEQIRKDIREFRTKSGVDKVIVLWTANTECFCDVVPGLNDTAENLLQAIERD-LEVS   257
Chrysemys_picta_bellii              --QVAQIRRDIQEFKATSGVDKVVVLWTANTERFCDVLPGVNDTGANLLQAIERG-LEVS   256
Ficedula_albicollis                 --QVEQIRRDIRDFRESSGVEKVIVLWTANTERFCDIVPGLNDTADNLLRAIERG-LEVS   256
Gallus_gallus                       --QVEQIRRDIRDFRASSGVDKVIVLWTANTERFCDVVPGLNDTADNLLRAIERG-LEVS   256
Cyprinus_carpio                     --QVEQIRRDIRDFRKKSGVDKVIVLWTANTERFCDVFPGVNDTAENLLNTIQTG-GEVS   256
Callorhinchus_milii                 --QIEQIRKDIQDFKTKSGVDKVIVLWTANTECFCDVVHGINDTAENLMKTVLQG-GEMS   256
Latimeria_chalumnae                 --QVEKIRKDIHDFKMQSCVDKVIVLWTANTERFCDVTPGVNDTAENLLKAIEKG-LEVS   218
Xenopus_laevis                      --QVQKIREDIQDFKRTSDVDKVIVLWTANTERFCDIIPGVNDTADNLLKAIENG-LEVS   256
Xenopus_tropicalis                  --QVQKIREDIQDFKKTSEVDKVIVLWTANTERFCDIIPGVNDTADNLLKAIENG-LEVS   256
                                         ::  ** .*     :: *:::****** : ::  * .     :   :

Caenorhabditis_elegans              PSNIFAVASILEGAHYINGSPQNTLVPGLIELAERHKVFVGGDDFKSGQTKFKSAFVDFL   328
Drosophila_melanogaster             PSTIFAMASIAEGCTYINGSPQNTFVPGLIQLAEEKNVFIAGDDFKSGQTKIKSVLVDFL   323
Culex_quinquefasciatus              PSTIFAMAAIAEGCIYINGSPQNTFVPGVIELAEHHGAFIAGDDFKSGQTKLKSVLVDFL   319
Aedes_aegypti                       PSTIFAMAAIAEGCIYINGSPQNTFVPGVVELAEHHGAFIAGDDFKSGQTKLKSVLVDFL   318
Petromyzon_marinus                  PSSLFAVASILEGCAYINGSPQNTFVPGVLELATQRGVFVGGDDFKSGQTKLKSVLVDFL   317
Apis_florea                         PSTVFAVAAALEGCTYINGSPQNTFVPGAIELAEKHKTFIGGDDFKSGQTKLKSVLVDFL   317
Strongylocentrotus_purpuratus       PSTLFAVASILEGVAYINGSPQNTFVPGVIEMAERRGVMIAGDDFKSGQTKIKSVLVDFL   314
Mus_musculus                        PSTLFAVASILEDCAFLNGSPQNTLVPGALELASQRHVFVGGDDFKSGQTKVKSVLVDFL   319
Saimiri_boliviensis                 PSTLFAVASILEGCAFLNGSPQNTLVPGALELACQRRVFVGGDDFKSGQTKVKSVLVDFL   247
Homo_sapiens                        PSTLFAVASILEGCAFLNGSPQNTLVPGALELAWQHRVFVGGDDFKSGQTKVKSVLVDFL   319
Bos_taurus                          PSTLFAVASILEGCAFLNGSPQNTLVPGALELAWQRRVFVGGDDFKSGQTKVKSVLVDFL   319
Canis_lupus                         PSTLFAVASILEGCAFLNGSPQNTLVPGALELARQRRVFVGGDDFKSGQTKVKSVLVDFL   319
Salmo_salar                         PSTLFAVASILEGCAYINGSPQNTFVPGAIELAVERNVFIAGDDFKSGQTKIKSVLVDFL   317
Gadus_morhua                        PSTLFAVASILEGCAYINGAPQNTFVPGAVELAVQKGVFIGGDDFKSGQTKIKSVLVDFL   277
Takifugu_rubripes                   PSSLFAVASILEGCAYINGSPQNTFVPGVIELAVQKGVFIGGDDFKSGQTKIKSVLVDFL   316
Tetraodon_nigroviridis              PSTLFAVASILEGCAYLNGSPQNTFVPGLIELAVQKRVFIGGDDFKSGQTKIKSVLVDFL   316
Xiphophorus_maculatus               PSTLFAVASILEDCAYINGSPQNTFVPGAVELAIQRGVFIGGDDFKSGQTKIKSVLVDFL   317
Oryzias_latipes                     PSTLFAVASILEGCAYINGAPQNTFVPGAVELAMQRGVFIAGDDFKSGQTKIKSVLVDFL   316
Gasterosteus_aculeatus              PSTLFAVASILEGCAYINGSPQNTFVPGAIELAVQNGVFIGGDDFKSGQTKVKSVLVDFL   316
Oreochromis_mossambicus_v1          ---------------------------------VQRGVFIGGDDFKSGQTKIKSVLVDFL   282
Oreochromis_mossambicus_v2          ---------------------------------VQRGVFIGGDDFKSGQTKIKSVLVDFL   311
Pundamilia_nyererei                 PSTLFAVASILEGCAYINGSPQNTFVPGAVELAMQRGVFIGGDDFKSGQTKIKSVFVDFL   317
Pseudotropheus_zebra                PSTLFAVASILEGCAYINGSPQNTFVPGAVELAMQRGVFIGGDDFKSGQTKIKSVFVDFL   317
Oreochromis_niloticus_v1            PSTLFAVASILEGCAYINGSPQNTFVPGAIELAMQRGVFIGGDDFKSGQTKIKSVLVDFL   317
Oreochromis_niloticus_v2            PSTLFAVASILEGCAYINGSPQNTFVPGAIELAMQRGVFIGGDDFKSGQTKIKSVLVDFL   346
Anolis_carolinensis                 PSTLFAVASILEGCPYINGSPQNTFVPGAVELAVQHQVFIAGDDFKSGQTKLKSVLVDFL   316
Anguilla_anguilla_v1                PSTLFAVASILEGCAYINGAPQNTFVPGAVELAVQKGVFIGGDDFKSGQTKIKSVLVDFL   316
Sarcophilus_harrisii                PSTLFAVASILEGCAYLNGAPQNTFVPGAIELAMQRRVYIGGDDFKSGQTKIKSVLVDFL   317
Chrysemys_picta_bellii              PSTLFAVASILEGCAYINGSPQNTFVPGAVELALQHHVFICGDDFKSGQTKLKSVLVDFL   316
Ficedula_albicollis                 PSTLFAVASILEGCAYINGSPQNTFVPGAVELAAQRRVFIGGDDFKSGQTKLKSVLVDFL   316
Gallus_gallus                       PSTLFAVASILEGCAYINGSPQNTFVPGAVELAAQRRVFICGDDFKSGQTKLKSVLVDFL   316
Cyprinus_carpio                     PSTMFAVASVLEGCAYINGSPQNTFVPGAVDLALQRGVFISGDDFKSGQTKLKSVLVDFL   316
Callorhinchus_milii                 PSSMFAVASILEGCAYINGSPQNTFVPGVIDLAMKHNVFIGGDDLKSGQTKIKSVLVDFL   316
Latimeria_chalumnae                 PSTLFAVASILEGCAYINGSPQNTFVPGAIELAIKHNVFIGGDDFKSGQTKIKSVLVDFL   278
Xenopus_laevis                      PSTMFAVASILEGCAYINGSPQNTFVPGAIELAVQNNVFIGGDDFKSGQTKIKSVLMDFL   316
Xenopus_tropicalis                  PSTMFAVASILEDCAYINGSPQNTFVPGAIELAIRHNVFIGGDDFKSGQTKIKSVLMDFL   316
                                                                      .. . : ***:******.**.::***

Caenorhabditis_elegans              VSSGMK-PESIVSYNHLGNNDGKNLSEARQFRSKEISKSSVVDDMVKSNQILFPDAKNPD   387
Drosophila_melanogaster             VGAGIK-PVSIASYNHLGNNDGKNLSAPQQFRSKEISKSNVVDDMVASNRLLYGPDEHPD   382
Culex_quinquefasciatus              VSAGIK-PVSIVSYNHLGNNDGRNLSAPQQFRSKEISKSNVVDDMVASNNILYKPEEHPD   378
Aedes_aegypti                       VSAGIK-PVSIVSYNHLGNNDGKNLSAPQQFRSKEISKSNVVDDMVASNNILYKPDEHPD   377
Petromyzon_marinus                  VSAGMKKTVSIVSYNHLGNNDGxxxxxxxxxxxxxxxxxxxxxxxxxxxxxxxxxxxxxx   377
Apis_florea                         VSAGIK-PVSIVSYNHLGNNDGYNLSAPQQFRSKEISKSNVVDDMVQSNKILYQSGEKPD   376
Strongylocentrotus_purpuratus       VSAGIK-PVSIVSYNHLGNNDGKNLSAPKQFRSKEISKSNVVDDMVESNPILYPDGKKPD   373
Mus_musculus                        IGSGLK-TMSIVSYNHLGNNDGQNLSAPLQFRSKEVTKSSVVDDMVHSNHVLYAPGERPD   378
Saimiri_boliviensis                 ISSGLK-TMSIVSYNHLGNNDGQNLSAPLQFRSKEVSKSNVVDDMVQSNPVLYMPGEEPD   306
Homo_sapiens                        IGSGLK-TMSIVSYNHLGNNDGENLSAPLQFRSKEVSKSNVVDDMVQSNPVLYTPGEEPD   378
Bos_taurus                          IGSGLK-TMSIVSYNHLGNNDGQNLSAPPQFRSKEVSKSSVVDDMVHSNPVLYSPGEQPD   378
Canis_lupus                         IGSGLK-TMSIVSYNHLGNNDGQNLSAPPQFRSKEVSKSSVVDDMVQSNPVLYAPGEEPD   378
Salmo_salar                         ISAGIK-PTSIVSYNHLGNNDGMNLSAPQQFRSKEISKSNVVDDMVNSNPILYRKGEKPD   376
Gadus_morhua                        VSAGIK-PTCIVSYNHLGNNDGMNLSAPQQFRSKEISKSNVVDDMVQSNPILFGPGEKPD   336
Takifugu_rubripes                   VSAGIK-PTSIVSYNHLGNNDGKNLSAPEQFRSKEISKSNVVDDMVQSNHILYKPGEKPD   375
Tetraodon_nigroviridis              VSAGIK-PTSIVSYNHLGNNDGKNLSAPQQFRSKEISKSNVVDDMVQSNHILYQPGEKPD   375
Xiphophorus_maculatus               VSAGIK-PTSIVSYNHLGNNDGKNLSAPQQFRSKEISKSNVVDDMVQSNPVLYKPGEKPD   376
Oryzias_latipes                     VSAGIK-PTSIVSYNHLGNNDGKNLSAPQQFRSKEISKSNVVDDIVQSNPILYKPGEKPD   375
Gasterosteus_aculeatus              VSSGIK-PTSIVSYNHLGNNDGKNLSAPQQFRSKEISKSNVVDDMVHSNSILYQPGEKPD   375
Oreochromis_mossambicus_v1          VSAGIK-PTSIVSYNHLGNNDGKNLSAPQQFRSKEISKSNVVDDMVQSNPILYEPGEKPD   341
Oreochromis_mossambicus_v2          VSAGIK-PTSIVSYNHLGNNDGKNLSAPQQFRSKEISKSNVVDDMVQSNPILYEPGEKPD   370
Pundamilia_nyererei                 VSAGIK-PTSIVSYNHLGNNDGKNLSAPQQFRSKEISKSNVVDDMVQSNPILYEPGEKPD   376
```

```
Pseudotropheus_zebra              VSAGIK-PTSIVSYNHLGNNDGKNLSAPQQFRSKEISKSNVVDDMVQSNPILFEPGEKPD  376
Oreochromis_niloticus_v1          VSAGIK-PTSIVSYNHLGNNDGKNLSAPQQFRSKEISKSNVVDDMVQSNPILYEPGEKPD  376
Oreochromis_niloticus_v2          VSAGIK-PTSIVSYNHLGNNDGKNLSAPQQFRSKEISKSNVVDDMVQSNPILYEPGEKPD  405
Anolis_carolinensis               VSSGIK-PVSIVSYNHLGNNDGRNLSAPAQFRSKEVSKSNVVDDMVDLNPVLYGPGERPD  375
Anguilla_anguilla_v1              VSAGIK-PAAIVSYNHLGNNDGKNLSAPQQFRSKEISKSNVVDDMVQSNPLLYGPGEKPD  375
Sarcophilus_harrisii              IGAGLK-TVSIVSYNHLGNNDGRNLSAPKQFHSKEVSKSNVVDDMVQSNPLLYGPGEEPD  376
Chrysemys_picta_bellii            ISAGLK-TVSIVSYNHLGNNDGKNLSAPQQFRSKEVSKSNVVDDMVQSNPLLYGPQEKPD  375
Ficedula_albicollis               VGAGLK-TKSIVSYNHLGNNDGKNLSAPQQFRSKEISKSNVVDDTVQANPVLYGPQDKPD  375
Gallus_gallus                     VGAGLK-TRSIVSYNHLGNNDGKNLSAPQQFRSKEISKSNVVDDTVQANPVLYGPQDKPD  375
Cyprinus_carpio                   ISAGIK-PTAIVSYNHLGNNDGLNLSAPQQFRSKEISKSNVVDDMVQSNPVLYRPGEKPD  375
Callorhinchus_milii               VSAGLK-TVSIVSYNHLGNNDGKNLSAPQQFRSKEISKSNVVDDMVQSNPILYKPSEKPD  375
Latimeria_chalumnae               ISAGLK-TVSIVSYNHLGNNDGKNLSAPQQFRSKEISKSNVVDDMVESNPILYGPREKPD  337
Xenopus_laevis                    VSAGLK-PVSIVSYNHLGNNDGKNLSAPQQFRSKEISKSNVVDDMVQSNPILYGPNEKPD  375
Xenopus_tropicalis                VNAGLK-PVSIVSYNHLGNNDGKNLSAPQQFRSKEISKSNVVDDMVESNPILYGPNEKPD  375
                                  : :*:*    .*.**********

Caenorhabditis_elegans            HCVVIKYVPYVADSKRAMDEYICSIFMGGKQTFVVHNTCEDSLLASPLIYDLAILTELAS  447
Drosophila_melanogaster           HVVVIKYVPYVGDSKRAMDEYTSEIMMGGHNTLVIHNTCEDSLLATPLILDLVILGELST  442
Culex_quinquefasciatus            HCVVIKYVPYVGDSKRAMDEYTSQIMLGGHNTLVIHNTCEDSLLASPLILDLAILGEFCS  438
Aedes_aegypti                     HCVVIKYVPYVGDSKRAMDEYTSQIMLGGHNTLIIHNTCEDSLLASPLILDLAILGELCS  437
Petromyzon_marinus                xxVVIKYVPYVGDSKRAMDEYTSEIMMGGLNTLVIHNTCEVTP--SPVL-----------  424
Apis_florea                       HCVVIKYVPYVGDSKRAMDEYTSEILLGGHNTIVVHNTCEDSLLASPIILDLVLLAEICS  436
Strongylocentrotus_purpuratus     HCVVIKYVPYVADSKRALDEYTSEIMMGGRNTISLHNTCEDSLLASPIILDLVILTELCQ  433
Mus_musculus                      HCVVIKYVPYVGDSKRALDEYTSELMLGGTNTLVLHNTCEDSLLAAPIMLDLVLLTELCQ  438
Saimiri_boliviensis               HCVVIKYVPYVGDSKRALDEYTSELMLGGTNTLVLHNTCEDSLLAAPIMLDLALLTELCQ  366
Homo_sapiens                      HCVVIKYVPYVGDSKRALDEYTSELMLGGTNTLVLHNTCEDSLLAAPIMLDLALLTELCQ  438
Bos_taurus                        HCVVIKYVPYVGDSKRALDEYTSELMLGGTNTLVLHNTCEDSLLAAPIMLDLALLTELCQ  438
Canis_lupus                       HCVVIKYVPYVGDSKRALDEYTSELMLGGTNTLVLHNTCEDSLLAAPIMLDLVLLTELCQ  438
Salmo_salar                       HCVVIKYVPYVGDSKRAMDEYTSEIMMGGTNTIAMHNTCEDSLLASPIILDLVLLTELCQ  436
Gadus_morhua                      HCVVIKYVPYVGDSKRAMDEYSSEIMMGGHNTIALHNTCEXXXLASPIILDLVMMTELCQ  396
Takifugu_rubripes                 HCVVIKYVPYVGDSKRAMDEYTSEIMMGGTNTIAMHNTCEDSLLASPIILDLVILTELCQ  435
Tetraodon_nigroviridis            HCVVIKYVPYVADSKRAMDEYTSEIMMGGTNTIAMHNTCEDSLLASPIILDLVILTELCQ  435
Xiphophorus_maculatus             HCVVIKYVPYVGDSKRAMDEYTSEIMMGGLNTIALHNTCEDSLLASPIILDLVILTELCQ  436
Oryzias_latipes                   HCVVIKYVPYVGDSKRAMDEYTSEIMMGGLNTIAMHNTCEDSLLASPIILDLVILTELCQ  435
Gasterosteus_aculeatus            HCVVIKYVPRVGDSKRAMDEYTSEIMMGGTNTIAMHNTCEDSLLATPIMLDLVILTELCQ  435
Oreochromis_mossambicus_v1        HCVVIKYVPYVGDSKRAMDEYTSEIMMGGINTIALHNTCEDSLLATPIILDLVMLTELCQ  401
Oreochromis_mossambicus_v2        HCVVIKYVPYVGDSKRAMDEYTSEIMMGGINTIALHNTCEDSLLATPIILDLVMLTELCQ  430
Pundamilia_nyererei               HCVVIKYVPYVGDSKRAMDEYTSEIMMGGINTIALHNTCEDSLLASPIILDLVMLTELCQ  436
Pseudotropheus_zebra              HCVVIKYVPYVGDSKRAMDEYTSEIMMGGINTIALHNTCEDSLLASPIILDLVMLTELCQ  436
Oreochromis_niloticus_v1          HCVVIKYVPYVGDSKRAMDEYTSEIMMGGINTIALHNTCEDSLLATPIILDLVMLTELCQ  436
Oreochromis_niloticus_v2          HCVVIKYVPYVGDSKRAMDEYTSEIMMGGINTIALHNTCEDSLLATPIILDLVMLTELCQ  465
Anolis_carolinensis               HCVVIKYVPYVGDSKRALDEYTSEIALGGTNTIVIHNVCEDSLLAAPIILDLVVLAELCQ  435
Anguilla_anguilla_v1              HCVVIKYVPYVGDSKRAMDEYTSEIMMGGTNTIALHNTCEDSLLASPIILDLVLLTELCQ  435
Sarcophilus_harrisii              HCVVIKYVPYVGDSKRALDEYTSEIMLGGTNTIVLHNTCEDSLLASPIILDLVILTELCQ  436
Chrysemys_picta_bellii            HCVVIKYVPYVGDSKRALDEYTSEIMMGGTNTIVIHNTCEDSLLASPIILDLAILTELCQ  435
Ficedula_albicollis               HCVVIKYVPYVGDSKRALDEYTSEIMMGGTNTIVIHNTCEDSLLASPIILDLAILTELCQ  435
Gallus_gallus                     HCVVIKYVPYVGDSKRALDEYTSEIMMGGTNTIVIHNTCEDSLLASPIILDLAILTELCQ  435
Cyprinus_carpio                   HCVVIKYVPYVGDSKRAMDEYTSEIMMGGTNTIALHNTCEDSLLASPIILDLVILTELCQ  435
Callorhinchus_milii               HCVVIKYVPYVGDSKRALDEYTSEIMMGGINTIVLHNTCEDSLLASPLILDLVILTELCQ  435
Latimeria_chalumnae               HCVVIKYVPYVGDSKRAMDEYTSEIMMGGTNTIVLHNTCEDSLLASPIILDLVILTELCQ  397
Xenopus_laevis                    HCVVIKYVPYVGDSKRAMDEYTSEIMMGGANTIVLHNTCEDSLLASPIILDLVLLTELCQ  435
Xenopus_tropicalis                HCVVIKYVPYVGDSKRAMDEYTSEIMMGGTNTIVLHNTCEDSLLASPIILDLVLLTELCQ  435
                                    ******* *.*****:***  ..: :** :*: :**.**     :*::

Caenorhabditis_elegans            RVSYKVD----DEYKPFHSVLSILSLLLKAPVVPPGTPISNAFMRQFSTLTKLVTALAGF  503
Drosophila_melanogaster           RIQLRNAEKESAPWVPFKPVLSLLSYLCKAPLVPQGSQVVNSLFRQRAAIENILRGCIGL  502
Culex_quinquefasciatus            RVQIKKKS--DTEYLPFRSVLSILSYLCKAPLVPQGTPVVNSLFRQRTAIENIMRACVGL  496
Aedes_aegypti                     RIQIKKKDESSAQYVPFRSVLSLLSYLCKAPLVPEGTPVVNSLFRQRTAIENILRACVGL  497
Petromyzon_marinus                ------------------------------------------------------------  424
Apis_florea                       RITFKIAD-TKDEFTGFHSVLSILSYLCKAPLVPRGTPIVNALFRQRAAIENILRACLAL  495
Strongylocentrotus_purpuratus     RIEFKSA--CQSEYQRFHSVLSILSYLCKAPLVPHNTPLVNALFKQRACMENIFRACVGL  491
Mus_musculus                      RVSFCTD--SDPEPQGFHTVLSLLSFLFKAPLVPPGSPVVNALFRQRSCIENIFRACVGL  496
Saimiri_boliviensis               RVSFCTD--ADPEPQTFHPVLSLLSFLFKAPLVPPGSPVINALFRQRSCIENILRACVGL  424
Homo_sapiens                      RVSFCTD--MDPEPQTFHPVLSLLSFLFKAPLVPPGSPVVNALFRQRSCIENILRACVGL  496
Bos_taurus                        RVSFCTD--VDPDPQSFHPVLSLLGFLFKAPLAPPGSPVVNALFRQRSCIENILRACVGL  496
Canis_lupus                       RVSFCTD--ADPEPQGFHSVLSLLSFLFKAPLVPPGSPVVNALFRQRSCIENILRACVGL  496
Salmo_salar                       RVCIRPQ--GSETFQSFHSVLAILSFMCKAPLVPPGAPLVNAYFRQRACIENIMRACLGL  494
Gadus_morhua                      RVRVRAP--GEQDFQSFHSVLALLSFFCKAPLMPPGAPLVNAFFRQRAAIENIMRACLGS  454
Takifugu_rubripes                 RVAVQLQ--GEEEFQSFHSVLALLAFLCKAPLVPPGSPVINAFFRQRACIENIMRACLGL  493
Tetraodon_nigroviridis            RVTVRPQ--GEEEFQSFHSVLALLAFLCKAPLVPPGTPVVNAFFRQRACIENIMRACLGL  493
Xiphophorus_maculatus             RVTVRPQ--GEEDFQSFHSVLALLSFLCKAPLVPSGTPVVNAFFRQRACIENIMRACLGL  494
Oryzias_latipes                   RVSIKPQ--GEEDFQSFHSVLALLSYLCKAPMVPSGTPVINAFFRQRACIENIMRACLGL  493
Gasterosteus_aculeatus            RITVRPQ--GEEDFQSFHSVLALLSFLCKAPLVPSGTPVINAFFRQRAAIENIMRACLGL  493
Oreochromis_mossambicus_v1        RVTVKPQ--GEESFQSFHSVLSLLSFLCKAPLVPSGTPVVNAFFRQRASIENIMRACLGL  459
Oreochromis_mossambicus_v2        RVTVKPQ--GEESFQSFHSVLSLLSFLCKAPLVPSGTPVVNAFFRQRASIENIMRACLGL  488
Pundamilia_nyererei               RVTVKPQ--GEENFQSFHSVLSLLSFLCKAPLVPSGTPVVNAFFRQRASIENIMRACLGL  494
Pseudotropheus_zebra              RVTVKPQ--GEENFQSFHSVLSLLSFLCKAPLVPSGTPVVNAFFRQRASIENIMRACLGL  494
Oreochromis_niloticus_v1          RVTVKPQ--GEESFQSFHSVLSLLSFLCKAPLVPSGTPVVNAFFRQRSSIENIMRACLGL  494
Oreochromis_niloticus_v2          RVTVKPQ--GEESFQSFHSVLSLLSFLCKAPLVPSGTPVVNAFFRQRSSIENIMRACLGL  523
Anolis_carolinensis               RIRVGIE--GEGDPQPLHSVLSLLSFLLKAPLVPSGAPVVNALFRQRAALENLLRACVGL  493
Anguilla_anguilla_v1              RISFCTE--SEPTFQTFHSVLSLLSYLLKAPLVPQGAPVVNSFFRQRACIENVLRACLGL  493
Sarcophilus_harrisii              RITFCTD--SDPHFQNFHSVLSILSFLCKAPLVPPGTPVVNALFRQRSCIENILRACVGL  494
Chrysemys_picta_bellii            RISFCTE--SDPDFQGFHSVLSILGFLCKAPLVPEGSPVVNALFRQRSCIENILRACVGL  493
Ficedula_albicollis               RITFCTE--ADPEFQGFHSVLSILAFLCKAPLVPEGTPVVNALFRQRSCIENILRYPGGP  493
```

```
Gallus_gallus                  RITFCTD--SDPEFQSFHSVLSIVAFLCKAPLVPEGTPVVNALFRQRSCIENILRACLGL  493
Cyprinus_carpio                RITFCTQ--DDPVFQGFHSVLSLLSFLCKAPLVPQGAPVVNAFFRQRACIENVMRACLGL  493
Callorhinchus_milii            RITFRTE--SDPEFQTFHSVLPILSYLCKAPLVPAGTPVINSLFRQRTCIENILRACLGL  493
Latimeria_chalumnae            RITFKTE--SDAEFQTFHSVLSILSFLCKAPLVPEGTPVINAFFRQRSCIENLFRACLGL  455
Xenopus_laevis                 RITFRTE--TDQEFQTFHSVLSILSFLCKAPLVPAGTPVINAFFRQRNCIENILRACLGL  493
Xenopus_tropicalis             RITFRTE--TDQEFQTFHSVLSILSFLCKAPLVPQGTPVVNALFRQRNCIENILRACLGL  493


Caenorhabditis_elegans         PSDTDMQIEFFTQLPAAKSKSQ--------------------------------------  525
Drosophila_melanogaster        PPISHMTLEQRFDFSTITNEPPL-KRVKILGQPCSVESVTNGKKLHANG-----HSNGSA  556
Culex_quinquefasciatus         PPVSHMSLEHRFNLNVSSVEQPPAKKSKVANS-SVTNG---TH-----------NSN---  538
Aedes_aegypti                  PPLSHMSLEHRFNVEVASTEP-PMKKGKISNE-TVTKPVSNGV-----------HTN---  541
Petromyzon_marinus             ------------------------------------------------------------  424
Apis_florea                    PPENNMLLEHKINFKNQL------------------------------------------  513
Strongylocentrotus_purpuratus  PPQNHMLLEHKLTNPSAMPELCMN-----G-----------------HSVNGKVLQNGNH  529
Mus_musculus                   PPQNHMLLEHKMERPGPGIKP-GE-----VVATSPLPCKKEPTP-ATNGCTG--DANGHP  547
Saimiri_boliviensis            PPQNHMLLEHKMERPGPSLKQVGP-----LAAARPVSNKKGPVPASTNGCTG--DANGHP  477
Homo_sapiens                   PPQNHMLLEHKMERPGPSLKRVGP-----VAATYPMLNKKGPVPAATNGCTG--DANGHL  549
Bos_taurus                     PPQNHMLLEHKMERPG--LKRVGP-----LATTSPVLCKKGSAPTAPNGCTG--DANGHS  547
Canis_lupus                    PPQNHMLLEHKMERPG--LKRVGP-----MVAACPVPCKKGPAPTAPNGCTG--DANGHS  547
Salmo_salar                    PPQNHMHLEHKLQRGFLPRHDNRI-----YDN---VAT--KMV-----------LSNGYH  533
Gadus_morhua                   PPQSNMQLEYKLQTGFLPQLTKGL-----ANG---TKAAA--------------------  486
Takifugu_rubripes              PPQNYMLLEHKLQKNFLSHMS--C-----NDK---VSALKKVA-----------LVNGTS  532
Tetraodon_nigroviridis         PPQNYMLLEHKLQKNFLSQVT--C-----NGE---VATLKKVA-----------LMNGTS  532
Xiphophorus_maculatus          PPQNHMLLEHKLQRNFLTAHSTYI-----NND---VATLKKAA-----------LANGNH  535
Oryzias_latipes                PPQNHMMLEHKLQRNFLSAHTTYV-----NSD---VAALKTFT-----------LPNGKH  534
Gasterosteus_aculeatus         PPQSHMLLEHKLQKNFLPQHTTYV-----NGD---VAALKKAS-----------LTNGVH  534
Oreochromis_mossambicus_v1     PPQNHMLLEHKLQRNFLPPHETCV-----NND---VASLKKVP-----------LVNGNH  500
Oreochromis_mossambicus_v2     PPQNHMLLEHKLQRNFLPPHETCV-----NND---VASLKKVP-----------LVNGNH  529
Pundamilia_nyererei            PPQNHMLLEHKLQRNFLPPHETCV-----NND---VASLKKVP-----------VVNGNH  535
Pseudotropheus_zebra           PPQNHMLLEHKLQRNFLPPHETCV-----NND---VASLKKVP-----------VVNGNH  535
Oreochromis_niloticus_v1       PPQNHMLLEHKLQRNFLPPHETCV-----NND---VASLKKVP-----------LVNGNH  535
Oreochromis_niloticus_v2       PPQNHMLLEHKLQRNFLPPHETCV-----NND---VASLKKVP-----------LVNGNH  564
Anolis_carolinensis            PPQNHMQLEYKTQQPWGCAKRDPS-----PKES-------PPPRLNGFACPP--ESNGHA  539
Anguilla_anguilla_v1           PPQGHMQLEHKMQRGGLTSSVG---------QICVTATTPKSAG---------VLKNGYH  535
Sarcophilus_harrisii           APQNHMLLEHKMERVSSEFRREGT-----EAPTCPVPMKKGVAASPPNGYVE--GTNGHQ  547
Chrysemys_picta_bellii         PPQNHMLLEHKMQRPCPSLKRV-------RLAECPVSLKRAAAHVNGYLCPG---SNGH-  542
Ficedula_albicollis            EC-RHPVPTVAPKAP-----QGGP-----GGSQCP--------------NPG---RPGR-  524
Gallus_gallus                  PPQNHMLLEHKMQRPPLGPKRCCP-----AGAACPITPKKAAPPTQLNGHPG---TPRP-  544
Cyprinus_carpio                PPQNHMQLEHKMKRSFMHSDENLM-----HHP---ILMNGKIEHINSYKSVKCIDINGHQ  545
Callorhinchus_milii            PPQNHMHLEHRMQRSFVTNRTAEH-------P---AAMLHKIPKLNNLSH-KNIVLNGH-  541
Latimeria_chalumnae            PCQNHMLLEHKMQKSFVPKKRAST-------S---V------------------------  481
Xenopus_laevis                 SPQNHMMLEHKMQRSFVSLKRPST-------V---CNPQPI-----SSKK-GNN-ANGFH  536
Xenopus_tropicalis             SPQNHMMLEHKMQRSFVSLKRPST-------V---CSPYPI-----SIKK-GNTVVNGFH  537


Caenorhabditis_elegans         ------------------------------------    525
Drosophila_melanogaster        KLATNGNGH---------------------------    565
Culex_quinquefasciatus         GTYTNGNGVHVDC-----------------------    551
Aedes_aegypti                  GTCTNGNGVHDIADY---------------------    556
Petromyzon_marinus             ------------------------------------    424
Apis_florea                    ------------------------------------    513
Strongylocentrotus_purpuratus  APERQPASKKAKLATTSSGFVNGNGVMENGSEVAAI    565
Mus_musculus                   QAPTPK--------------------LSTA------    557
Saimiri_boliviensis            VEE-PQ--------------------MPT-------    485
Homo_sapiens                   QEEPP---------------------MPTT------    558
Bos_taurus                     QAEAPQ--------------------MPTT------    557
Canis_lupus                    QAEAPQ--------------------MPTT------    557
Salmo_salar                    A--SQQNGVCAHVVKEAT-------IG---------    551
Gadus_morhua                   ------------------------------------    486
Takifugu_rubripes              K--PLTNGFCTLSDLHTD-------LLTTDIEIFR-    558
Tetraodon_nigroviridis         K--PLTNGFCT-------------------------    541
Xiphophorus_maculatus          TPVPLKNGIYTHKSDTAC-------AM---------    555
Oryzias_latipes                VP----------------------------------    536
Gasterosteus_aculeatus         I--PLTNGIHTHLDDATH-------AL---------    552
Oreochromis_mossambicus_v1     I--PLTNGVYAHMDHTAC-------AL---------    518
Oreochromis_mossambicus_v2     I--PLTNGVYAHMDHTAC-------AL---------    547
Pundamilia_nyererei            I--PLTNGVYAHMDHTAC-------AL---------    553
Pseudotropheus_zebra           I--PLTNGVYAHMDHTAC-------AL---------    553
Oreochromis_niloticus_v1       I--PLTNGVYAHMDHTAC-------AL---------    553
Oreochromis_niloticus_v2       I--PLTNGVYAHMDHTAC-------AL---------    582
Anolis_carolinensis            EPC---------LGLQN-------------------    547
Anguilla_anguilla_v1           VPV-L-NGVQ-HNGFETH--------R----------    551
Sarcophilus_harrisii           HAS-AS------------------------------    552
Chrysemys_picta_bellii         -GT-SQA-----------------------------    547
Ficedula_albicollis            -PV-LVGGSQ-CHQWVPV-------RAGG-------    543
Gallus_gallus                  -PL-HIDGAE--------------------------    552
Cyprinus_carpio                KIR-HT--VS--------------------------    552
Callorhinchus_milii            -----------TNGY---------------------    545
Latimeria_chalumnae            ------------------------------------    481
Xenopus_laevis                 LPG-ISKGLSQSNGLGKN-------VINSDIEIEN-    563
Xenopus_tropicalis             PPS-ISNGLSHSNGLGKT-------MISSDIEIEN-    564
```

Supplementary Figure 1 Cross-species amino acid alignment of MIPS sequences. The inclusion of intron 5/6 in tilapia $MIPS_{(1)}$ (v2) is indicated in red. A potential 3 amino acid repeat in *O. mossambicus* MIPS is indicated in pink. * designates fully conserved residues across all sequences, . and : indicate respective weak and strong conservation of amino acids with similar properties. S, T and Y amino acids highlighted indicate potential phosphorylation sites in eel and tilapia sequences (Expasy NetPhos predictor). Amino acids highlighted in blue in the *Xenopus* sequence indicate highly conserved motifs involved in "core structure and function" and found in all eukaryotic species. Amino acids highlighted in green in the *Xenopus* sequence indicate conserved amino acids found at the enzyme active site. Amino acids in the human sequence highlighted in orange indicate the epitope for the Abnova polyclonal, in magenta indicate the epitope for the Abcam polyclonal and underlined in magenta, the Santa Cruz Biotechnology monoclonal anti-human ISYNA1 antibodies used in this study. Accession numbers for all species are presented in Table 2.
