# Supplementary material for: Myo-inositol phosphate synthase expression in the European eel (Anguilla anguilla) and Nile tilapia (Oreochromis niloticus): effect of seawater acclimation
Source: Am J Physiol Regul Integr Comp Physiol. 2016 Jun 1;311(2):R287–98. doi: 10.1152/ajpregu.00056.2016 (PMC5008666; doi:10.1152/ajpregu.00056.2016)
Supplement: Supplemental Table S1 [file Supplemental_Table_S1.pdf]

Supplemental Table S1. Cross-species MIPS amino acid sequence homologies derived from Clustal Ω alignment.

MIP synthase % amino acid homology	Oreochromis niloticus	Oreochromis mossambicus	Pundamilia nyererei	Pseudotropheus zebra	Xiphophorus maculatus	Oryzias latipes	Gasterosteus aculeatus	Takifugu rubripes	Tetraodon nigroviridis	Salmo salar	Gadus morhua	Cyprinus carpio	Anguilla anguilla	Latimeria chalumnae	Petromyzon marinus	Callorhinchus milii	Xenopus laevis
Oreochromis niloticus	100																
Oreochromis mossambicus	97.49	100															
Pundamilia nyererei	97.47	95.75	100														
Pseudotropheus zebra	97.29	95.56	99.82	100													
Xiphophorus maculatus	89.33	87.26	88.79	88.61	100												
Oryzias latipes	87.69	83.59	87.13	86.94	89.55	100											
Gasterosteus aculeatus	86.78	85.14	85.69	85.51	85.33	86.57	100										
Takifugu rubripes	82.82	81.27	82.28	82.1	81.08	83.96	83.15	100									
Tetraodon nigroviridis	84.84	81.85	83.73	83.55	85.03	84.33	85.03	91.5	100								
Salmo salar	78.95	76.83	78.4	78.22	78.4	80.78	77.68	76.59	78.93	100							
Gadus morhua	81.48	74.28	81.28	81.48	81.28	80.66	80.66	79.22	80.66	80.04	100						
Cyprinus carpio	76.27	74.71	76.45	76.27	76.09	76.87	75.36	73.37	75.05	75.32	77.16	100					
Anguilla anguilla	76.77	74.71	76.41	76.23	76.04	77.99	74.77	73.87	76.16	74.05	78.19	76.59	100				
Latimeria chalumnae	79.83	71.52	79	78.79	78.38	78.17	78.79	78.59	79.21	77.13	74.43	80.67	78.79	100			
Petromyzon marinus	63.92	54.72	64.39	64.39	63.21	61.32	60.85	62.97	62.26	62.97	59.2	62.74	63.44	60.38	100		
Callorhinchus milii	69.72	66.6	69.54	69.36	71.01	71.27	69.91	69.17	70.43	68.44	70.78	71.01	72.29	81.5	62.26	100	
Xenopus laevis	72.33	70.08	71.79	71.61	72.07	73.32	70.65	70.61	73.75	70.42	73.46	71.38	73.32	87.32	64.62	77.25	100
Xenopus tropicalis	71.43	69.11	71.07	70.89	71.17	72.2	69.38	69.18	72.27	70.05	72.43	71.01	73.14	86.49	64.15	77.06	93.43
Chrysemys picta bellii	70.57	66.99	70.93	70.75	71.48	71.64	70.2	70.57	71.53	69.47	72.02	72.58	74.41	83.37	64.86	74.5	78.25
Anolis carolinensis	70.02	67.18	70.38	70.2	70.93	71.27	70.75	68.37	69.69	69.1	72.84	70.02	70.02	76.72	63.21	69.36	71.85
Ficedula albicollis	69.98	65.83	70.17	69.98	71.45	70.52	69.06	69.43	70.98	68.32	70.37	71.09	71.09	79.83	66.51	72.38	74.95
Gallus gallus	72.1	69.31	72.46	72.28	72.46	72.95	71.74	71.2	73.75	70.24	72.84	73.19	72.6	83.37	66.98	74.68	76.63
Sarcophilus harrisii	68.66	65.64	68.48	68.3	68.84	69.22	67.21	66.3	68.58	67.15	70.78	70.47	70.05	79	61.56	72.48	72.1
Mus musculus	65.1	62.55	64.74	64.56	66.13	65.49	65.04	63.38	64.88	65.15	66.46	66.67	66.24	72.97	61.08	64.77	65.17
Bos taurus	65.46	62.93	65.28	65.1	65.77	65.86	65.76	63.38	66.36	65.52	68.31	66.3	66.97	72.56	62.03	65.14	66.25
Canis lupus	66	63.51	65.82	65.64	65.95	66.04	65.58	63.55	65.99	65.34	68.31	66.85	67.88	72.77	61.32	66.06	66.79
Homo sapiens	65.1	62.55	64.92	64.74	64.86	65.86	65.22	62.72	65.43	64.61	67.28	66.49	67.88	73.39	61.79	67.52	67.74
Saimiri boliviensis	64.12	57.32	63.71	63.51	63.51	63.51	64.12	62.68	62.47	63.71	59.79	65.36	64.95	65.7	49.29	64.74	65.57
Apis florea	68.42	61.21	68.23	68.03	67.84	66.86	66.67	64.72	66.86	65.69	62.96	66.28	68.03	69.85	55.42	67.84	69.01
Drosophila melanogaster	58.95	56.37	58.59	58.41	58.02	58.58	58.7	56.81	58.41	58.44	60.91	57.25	58.44	63.83	56.84	59.45	57.37
Culex quinquefasciatus	59.89	57.14	59.71	59.53	59.89	60.45	59.53	59.71	59.89	59.17	60.49	57.71	58.98	64.45	56.37	61.65	59.53
Aedes aegypti	60.22	58.3	60.22	60.04	59.82	61.57	60.87	60.43	61.18	59.17	61.32	59.06	60.62	64.66	55.66	61.1	59.17
Caenorhabditis elegans	51.43	45.17	51.24	51.43	50.67	50.48	49.52	50.1	51.05	49.14	51.23	49.33	50.29	54.89	49.76	50.67	51.24
Strongylocentrotus purpuratus	67.27	64.29	66.91	66.73	67.21	68.28	65.4	65.95	67.84	64.97	67.28	64.31	65.15	72.77	60.61	65.87	64.83

MIP synthase % amino acid homology	Xenopus tropicalis	Chrysemys picta bellii	Anolis carolinensis	Ficedula albicollis	Gallus gallus	Sarcophilus harrisii	Mus musculus	Bos taurus	Canis lupus	Homo sapiens	Saimiri boliviensis	Apis florea	Drosophila melanogaster	Culex quinquefasciatus	Aedes aegypti	Caenorhabditis elegans	Strongylocentrotus purpuratus
Oreochromis niloticus																	
Oreochromis mossambicus																	
Pundamilia nyererei																	
Pseudotropheus zebra																	
Xiphophorus maculatus																	
Oryzias latipes																	
Gasterosteus aculeatus																	
Takifugu rubripes																	
Tetraodon nigroviridis																	
Salmo salar																	
Gadus morhua																	
Cyprinus carpio																	
Anguilla anguilla																	
Latimeria chalumnae																	
Petromyzon marinus																	
Callorhinchus milii																	
Xenopus laevis																	
Xenopus tropicalis	100																
Chrysemys picta bellii	78.06	100															
Anolis carolinensis	72.21	74.59	100														
Ficedula albicollis	74.77	79.93	72.93	100													
Gallus gallus	76.81	83.55	75.87	86.19	100												
Sarcophilus harrisii	72.1	75.69	71.3	73.66	76.81	100											
Mus musculus	65.89	70.57	68.19	68.14	71.74	74.46	100										
Bos taurus	66.97	72.76	70.02	69.98	74.46	77.17	87.07	100									
Canis lupus	67.33	72.58	70.38	70.17	74.28	78.62	87.61	93.72	100								
Homo sapiens	68.64	72.76	70.02	69.98	74.82	78.08	87.43	91.38	91.56	100							
Saimiri boliviensis	66.19	68.25	66.39	65.57	70.93	74.85	83.3	85.36	86.8	90.72	100						
Apis florea	69.01	66.28	67.45	64.13	66.86	65.11	61.6	61.6	62.38	62.18	56.08	100					
Drosophila melanogaster	56.74	58.87	59.23	57.27	58.88	59.42	56.55	57.63	57.27	57.71	57.32	61.01	100				
Culex quinquefasciatus	59.89	59.6	59.05	58.2	58.8	58.8	56.99	57.71	57.17	57.53	57.11	64.91	67.51	100			
Aedes aegypti	58.99	61.97	59.42	59.12	59.96	59.06	56.12	57.19	57.01	56.83	56.49	63.94	68.35	85.66	100		
Caenorhabditis elegans	51.81	49.33	50.1	49.9	50.48	49.14	49.52	49.9	49.71	50.48	49.07	52.24	48.19	50.48	49.71	100	
Strongylocentrotus purpuratus	65.07	65.45	63.62	63.72	65.58	64.86	58.35	59.25	59.43	59.32	58.35	63.74	55.75	58.08	57.37	54.48	100
